# SPACA6P-AS: a trailblazer in breast cancer pathobiology and therapeutics

**DOI:** 10.1007/s10565-024-09870-9

**Published:** 2024-06-26

**Authors:** Wenjie Feng, Yiling Jiang, Lijun Zeng, Yuhan Ouyang, Hailong Li, Yuanbin Tang, Lunqi Luo, Lianjie Ouyang, Liming Xie, Yeru Tan, Yuehua Li

**Affiliations:** 1 Department of Oncology, the First Affiliated Hospital, Hengyang Medical School, University of South China, Hengyang, Hunan People’s Republic of China; 2 Department of Pathology, Changde Hospital, Xiangya School of Medicine, Central South University, the First People’s Hospital of Changde City, Changde, Hunan People’s Republic of China

**Keywords:** Breast cancer, Long noncoding RNA SPACA6P-AS, Gene expression, Enrichment analysis, Immune infiltration, In vitro and in vivo experiments

## Abstract

**Objective:**

The primary objective of this investigation is to delve into the involvement of the long noncoding RNA (lncRNA) SPACA6P-AS in breast cancer (BC) development, focusing on its expression pattern, association with clinical-pathological features, impact on prognosis, as well as its molecular and immunological implications.

**Methods:**

Bioinformatics analysis was conducted utilizing RNA sequencing data of 1083 BC patients from the TCGA database. Functional exploration of SPACA6P-AS was carried out through the construction of survival curves, GO and KEGG enrichment analysis, and single-sample gene set enrichment analysis (ssGSEA). Furthermore, its functionality was validated through in vitro cell experiments and in vivo nude mouse model experiments.

**Results:**

SPACA6P-AS showed a remarkable increase in expression levels in BC tissues (*p* < 0.001) and demonstrated a close relationship to poor prognosis (overall survival HR = 1.616, progression-free interval HR = 1.40, disease-specific survival HR = 1.54). Enrichment analysis revealed that SPACA6P-AS could impact biological functions such as protease regulation, endopeptidase inhibitor activity, taste receptor activity, taste transduction, and maturity-onset diabetes of the young pathway. ssGSEA analysis indicated a negative correlation between SPACA6P-AS expression and immune cell infiltration like dendritic cells and neutrophils, while a positive correlation was observed with central memory T cells and T helper 2 cells. Results from in vitro and in vivo experiments illustrated that silencing SPACA6P-AS significantly inhibited the proliferation, migration, and invasion capabilities of BC cells. In vitro experiments also highlighted that dendritic cells with silenced SPACA6P-AS exhibited enhanced capabilities in promoting the proliferation of autologous CD3 + T cells and cytokine secretion. These discoveries elucidate the potential multifaceted roles of SPACA6P-AS in BC, including its potential involvement in modulating immune cell infiltration in the tumor microenvironment.

**Conclusion:**

The high expression of lncRNA SPACA6P-AS in BC is closely linked to poor prognosis and may facilitate tumor progression by influencing specific biological processes, signaling pathways, and the immune microenvironment. The regulatory role of SPACA6P-AS positions it as a prospective biomarker and target for therapeutic approaches for BC diagnosis and intervention.

**Supplementary Information:**

The online version contains supplementary material available at 10.1007/s10565-024-09870-9.

## Introduction

Breast cancer (BC) represents a widely recognized type of cancer that predominantly affects women on a global scale and is a leading factor in cancer-related deaths affecting the female population (Martuszewski et al. 2020). It accounts for 15% of all new cancer cases in females (Siegel et al. 2022; Metcalfe et al. 2023; Lai et al. 2021). The etiology of BC is multifaceted, encompassing genetic, environmental, and lifestyle factors (Zou et al. 2022; Najjar and Allison 2022; Howard and Olopade 2021). Despite recent advancements in treatment, the complexity of BC tumor formation and progression mechanisms continues to challenge effective treatment (Han et al. 2023). Typical BC treatments include surgery, radiotherapy, chemotherapy, and endocrine therapy. However, the tumor's high heterogeneity leads to significant variability in treatment outcomes (Sekine et al. 2021; Heitz et al. 2023). Existing biomarkers such as CEA, CA125, and CA153 aid in early detection and prognosis but are limited in precision and specificity (Mirabelli and Incoronato 2013; Liang et al. 2023; Qin et al. 2021). Therefore, exploring more effective biomarkers and therapeutic targets is crucial for enhancing BC diagnosis and treatment efficacy.

Long noncoding RNAs (lncRNAs), RNA molecules exceeding 200 nucleotides, play critical roles in gene expression regulation and disease progression despite not encoding proteins (Nojima and Proudfoot 2022; Chen et al. 2022). LncRNA research has become a focus in oncology (Cervena et al. 2022; He et al. 2024), with these molecules implicated in various mechanisms of tumor initiation and progression, such as gene transcription regulation, mRNA stability and translation, cell cycle, and apoptosis (Shaath et al. 2022; Li and Wang 2023; Liu et al. 2022). In BC, abnormal expression of specific lncRNAs correlates with tumor cell proliferation, migration, and invasion (Li et al. 2023), highlighting their potential as new therapeutic targets or prognostic markers (Tan et al. 2021; Park et al. 2021; Liu et al. 2021; Hashemi et al. 2022).

LncRNA SPACA6P-AS, located at 19q13.41 and recently noted in tumor research (Xu et al. 2021), exhibits aberrant expression in various cancers, including liver cancer, suggesting its significant role in tumorigenesis and progression (Xu et al. 2021). Studies indicate SPACA6P-AS influences the expression and function of several molecules, including miR-125a, by forming novel ceRNA regulatory networks (Di Palo et al. 2020). Prognostic analysis in BC patients reveals a seven-lncRNA signature, including SPACA6P-AS, closely related to survival rates (Xu et al. 2021; Powell et al. 2022; Ciabattoni et al. 2022; Li et al. 2022a). Nevertheless, the specific functions and operational mechanisms of SPACA6P-AS in BC remain underexplored (Xu et al. 2021).

Utilizing public databases such as The Cancer Genome Atlas (TCGA), we comprehensively analyzed SPACA6P-AS's genomic alterations and functional networks, including its potential role in tumor immunity. Our research encompasses bioinformatics analysis and in vitro and in vivo experiments to assess SPACA6P-AS's role and mechanisms in BC. These studies contribute to a deeper understanding of SPACA6P-AS's role in BC progression, particularly in immune microenvironment modulation and cellular behavior regulation.

This study aims to unveil SPACA6P-AS's role in BC by examining its implications on immune cell infiltration and the tumor microenvironment. We aim to identify new therapeutic strategies and biomarkers through a comprehensive evaluation of SPACA6P-AS's expression patterns, correlations with clinicopathological features, and effects on BC patient prognosis. Operating as fresh biomarker and prospective therapeutic aim, SPACA6P-AS research could pave new pathways for diagnosis and treatment, significantly improving survival rates and quality of life for BC patients. Our work may provide more direct evidence for hypotheses regarding SPACA6P-AS expression as a plausible therapeutic target or predictive biomarker within the scope of risk profiling.

## Materials and methods

### RNA sequencing and clinical data analysis from TCGA database

RNA sequencing data of tumor tissues from 1083 BC patients and 113 adjacent normal tissue samples were sourced from TCGA database, along with clinical data. Furthermore, the Genotype-Tissue Expression (GTEx) database provided 179 normal breast tissue samples. Utilization of the DESeq2 R package (version 1.20.0, Bioconductor) enabled the performance of data analysis following the guidelines in PMID: 25516281. The expression levels of the lncRNA SPACA6P-AS were ranked across all patient samples to establish the median expression level. Based on this median expression level, two distinct groups were formulated within the BC patient cohort: the high expression group (*N* = 542) and the low expression group (*N* = 541). A comparison of HTSeq-counts data between the high and low expression groups of lncRNA SPACA6P-AS was executed. The threshold was established at |log2 Fold Change (FC)|> 1.5, and adjusted p-values were calculated utilizing the Benjamini–Hochberg approach, considering an adjusted *p* < 0.05 as significant. Identification of differentially expressed genes (DEGs) was carried out using the R package limma to investigate biological variances between the high and low expression groups.

### Construction and evaluation of nomograms

Through the utilization of multivariate Cox regression models, prognostic nomograms were formulated specifically for patients diagnosed with BC using the 'survival' and 'rms' packages in R software (version 4.2.0). The total prognostic score for every individual identified with BC was calculated considering clinical parameters such as age, pathological staging, tumor size, etc. Calibration plots of the nomograms were used to assess prediction accuracy, while the concordance index (C-index) and bootstrapping with 1000 resamples were employed to evaluate the model's discriminative ability.

### Enrichment analysis

Enrichment analysis of Gene Ontology (GO) and Kyoto Encyclopedia of Genes and Genomes (KEGG) pathways was carried out employing the clusterProfiler R package (version 3.14.3), involving over-representation analysis (ORA) (Yu et al. 2012). The objective of this stage was to pinpoint key biological processes (BP) and signaling pathways connected to the differential expression of SPACA6P-AS.

### Gene set enrichment analysis (GSEA)

The GSEA software (version 3.0) was employed for the implementation of the GSEA analysis. DEGs between high and low SPACA6P-AS expression groups were subjected to 1000 permutation analyses to obtain normalized enrichment scores (NES), with NOM p-values below 0.05 considered significant.

### ssGSEA immune infiltration analysis

The GSVA R package (version 1.34.0) was employed to perform single-sample gene set enrichment analysis (ssGSEA) on 24 types of tumor-infiltrating immune cells in order to quantitate immune infiltration in BC tumor samples (Bindea et al. 2013). This analysis was conducted based on 509 gene features linked to various immune cells, including natural killer cells (NK cells), tumor-infiltrating neutrophils (TIN), CD56 bright/dim NK cells, and others. The correlation between immune cell infiltration and lncRNA SPACA6P-AS expression was investigated through Spearman correlation analysis.

### Cell culture

In this study, we used two human BC cell lines, MDA-MB-231 (ATCC, CRM-HTB-26™) and MCF-7 (ATCC, HTB-22), to simulate different subtypes of BC. Cellular growth occurred in Dulbecco's Modified Eagle Medium (DMEM) containing 10% fetal bovine serum (FBS, Gibco, 12484028) and 1% penicillin–streptomycin (Gibco, 15140–122), providing essential nutrients and preventing microbial contamination. Cultures were upheld at 37 °C in a 5% CO_2_ incubator (Forma™ Steri-Cult™, 3308E) to replicate physiological conditions. There was a renewal of the medium bi-daily, and cells were passaged upon reaching approximately 80% confluence to maintain vitality and proliferative capacity.

For evaluating the impact of lncRNA SPACA6P-AS on immune cells, tumor cell lysates were prepared as follows: Cell suspensions of MDA-MB-231 were exposed to six rapid freeze–thaw cycles in liquid nitrogen and subsequently treated in a 37 °C water bath. Subsequent ultrasonication lasting 15 s facilitated the extraction of tumor antigens from lysed cancer cells. The cell debris was removed from the lysates by centrifugation at 1500 rpm for 15 min at 4 °C. Subsequently, the supernatant was passed through a 0.2 µm filter from Millipore (SLGP033RB). Measurement of protein quantities in the lysates was carried out by employing the Bio-Rad Bradford protein assay kit (500–0006).

### Cell transfection

Human BC cell line MDA-MB-231 cells were transfected and grouped as follows: sh-NC group (transfected with the sequence 5'-GGGUGAACUCACGUCAGAA-3'), sh-SPACA6P-AS-#1 group (transfected with the sequence 5'-GAGATAGGCACAGAGACATTC-3'), and sh-SPACA6P-AS-#2 group (transfected with the sequence 5'-GGCACAGAGACATTCTGAGAG-3'). These groups were also utilized in establishing a mouse xenograft model. Subsequent cell experiments were conducted using the transfection of sh-SPACA6P-AS with better transfection efficiency in MDA-MB-231 cells, MCF-7 cell line, and dendritic cells (DCs). The transfection procedures adhered to the manufacturer's guidelines of Lipofectamine 2000 (Invitrogen, part number: 11668019) to ensure maximal transfection efficiency and minimal cellular toxicity. After 48 h post-transfection, the silencing efficiency of the sh-SPACA6P-AS sequence was assessed using RT-qPCR (A31673, Applied Biosystems™). The specific primer sequences are detailed in Table S1.

### Cell proliferation assay

The impact of lncRNA SPACA6P-AS on the proliferation capability of BC cells was carried out using the CCK-8 assay. After transfection, the cells received treatment involving the CCK-8 reagent (ab228554, Abcam, USA) at 24, 48, and 72 h and incubated for a specified duration adhering to the manufacturer's prescribed guidance. Subsequently, the absorbance values post-reaction were measured using a spectrophotometer (1681150, Bio‐Rad, USA) at a wavelength of 450nm to quantify the proliferative activity of the cells (Nie et al. 2024).

### Migration and invasion experiments

The involvement of lncRNA SPACA6P-AS in BC cell migration and invasion was analyzed using Scratch and Transwell invasion assays. For the scratch assay, cultured cells in 6-well plates (Corning, 3516) were allowed to grow until reaching 90% confluency. To initiate the experiment, a scratch of approximately 1 mm in size was meticulously made using a 200 µL pipette tip. Cultivated cells were subjected to serum-deprived conditions for 24 to 48 h, and the healing process was observed and photographed using a phase contrast microscope (Olympus, CKX53). For the Transwell invasion assay, the porous membrane of the upper chamber (Corning, 3422) was coated with Matrigel (BD Biosciences, 354234). The upper compartment received the processed cell suspension, while the lower compartment was filled with DMEM supplemented with 10% FBS. Post 24 h of incubation, non-migratory cells in the upper chamber were eliminated using a cotton swab, and the migrated cells that traversed the membrane were subsequently fixed with 4% paraformaldehyde (Catalog: G1101, Servicebio) and stained with 0.1% crystal violet (C0775, Sigma-Aldrich, St. Louis, MO, USA) for the purpose of cell enumeration under a microscope (Li et al. 2024; Katz et al. 2017).

### Construction of a nude mouse animal model

In this experiment, from Beijing Vital River Laboratory Animal Technology Co., Ltd. (catalog number: 401, Beijing, China), 18 healthy female BALB/c Nude mice were procured. Aseptic housing was provided for the mice in a designated animal facility, where conditions involved maintaining a humidity level between 60 and 65% alongside a temperature spectrum ranging from 20 to 25°C. Following a week of acclimation nourishment, the mice were randomly allocated to three separate groups for the study following the end of the adaptation period. The group size was fixed at 6 mice, which were then transfected with sh-lncRNA SPACA6P-AS#1, sh-lncRNA SPACA6P-AS#2, and sh-NC MDA-MB-231 cells, respectively. Health assessments were conducted on the mice before the commencement of the experiment. Each mouse received approximately 1 × 10^6^ cells via subcutaneous injection in the flank (Sang et al. 2018). Post-injection, the mice's health status and tumor growth were regularly monitored and recorded. Tumor size was measured with a ruler, calculated using the formula volume = length × width^2^/2, and mice were euthanized two weeks after injection.

### Hematoxylin and eosin (H&E) staining and immunohistochemistry

Nude mouse xenograft tumor tissues were fixed with 4% formaldehyde solution, dehydrated and cleared with xylene (Catalog Number: 214736, Fisher Scientific) and a series of ethanol concentrations (Catalog Number: BP2818-4, Fisher Scientific). Subsequently, the tissues were encased in paraffin and sliced into sections measuring 4 µm in thickness. The slices were melted in an oven at 56 °C, deparaffinized twice with xylene, and sequentially immersed in 100%, 95%, 90%, and 75% ethanol. Subsequent staining steps included immersion in hematoxylin (Catalog Number: 51275, Sigma) for 3 s, rinsing in tap water for 1 min, staining with 1% eosin Y (Catalog Number: 318906, Sigma) for 5 min, followed by a repeated dehydration process (70%, 80%, 90%, 95%, 100% ethanol). Coverslips were sealed with neutral gum (Catalog Number: G8593, Beijing Solarbio Science and Technology Co., Ltd.), air-dried, and observed under a microscope for recording (Wu et al. 2023; Yang et al. 2023).

The slides were deparaffinized at 58 °C for 30 min, undergo antigen retrieval with citrate buffer (pH 6.0, Catalog Number: CL-K943, Shanghai Yihai), followed by blocking of endogenous peroxidase with 3% hydrogen peroxide (Catalog Number: H1009, Sigma-Aldrich). Subsequently, non-specific binding was blocked using 5% BSA (Catalog Number: A9647, Sigma-Aldrich). The slides were then incubated with Ki-67 antibody (Catalog Number: ab15580, Abcam) overnight at 4 °C. The next day, staining was performed using secondary antibody and DAB chromogen (Catalog Number: K5007, DAKO), followed by counterstaining of cell nuclei with hematoxylin and mounting with mounting medium. Cell counting under an optical microscope (Optika, XDS-3, Ponteranica, Italy) was conducted by randomly selecting 5 fields per slide and counting 100 cells per field to determine the percentage of Ki-67 positive cells. Immunohistochemistry specificity was confirmed by performing positive control (BC tumor tissues) and negative control (non-immune serum without primary antibody) (Bouraoui et al. 2018; Vieira Costa et al. 2024).

### Obtaining peripheral blood mononuclear cells and cultivating DCs

Peripheral blood was collected from healthy donors (*n* = 3, 2 males, 1 female, average age 34.2 ± 2.3 years) using sterile heparinized Falcon tubes (BD Biosciences, Cat. No. 352054). The separation of peripheral blood mononuclear cells (PBMCs) from blood samples was carried out utilizing Ficoll gradient separation method (GE Healthcare, Cat. No. 17144002). Monocytes were subsequently separated from PBMCs through adherence to a plastic surface. To achieve this, PBMCs were seeded at a concentration of 5 × 10^6^ cells per milliliter in serum-free RPMI-1640 medium (Gibco, Cat. No. 11875–093) in 6-well plates (Corning, Cat. No. 3516). Following incubating at 37 °C for 2 h, non-adherent cells were discarded, and culturing of the adherent cells was conducted in a medium supplemented with 50 µM 2-mercaptoethanol (Sigma-Aldrich, Cat. No. M6250), 20 ng/mL recombinant human IL-4 (rhIL-4, PeproTech, Cat. No. 200–04), and 40 ng/mL recombinant human GM-CSF (rhGM-CSF, PeproTech, Cat. No. 300–03). Fresh medium enriched with rhGM-CSF and rhIL-4 was introduced to the remaining culture halves on days 2 and 4. On day 6, immature dendritic cells (iDCs) were harvested, and the culture received an addition of 80 µg/mL of mixed lysate from human BC cell lines. After 5 h of incubation, 100 ng/mL of lipopolysaccharide (LPS, Sigma-Aldrich, Cat. No. L4391) was incorporated into the culture medium. Mature dendritic cells (mDCs) loaded with tumor cell lysates were obtained after a 24-h incubation at 37 °C.

### Identification of the morphology and phenotypic characteristics of DCs

The morphology of mononuclear cells and DCs was observed and captured through an inverted optical microscope (Optika, XDS-3, Ponteranica, Italy). To investigate the phenotypic features of iDCs, mDCs, and mDCs with silenced SPACA6P-AS, surface markers including CD11c (anti-CD11c-FITC, BioLegend, catalog number: 301306), HLA-DR (anti-HLA-DR-APC, BioLegend, catalog number: 307606), CD40 (anti-CD40-CF Blue, BioLegend, catalog number: 33432), and CD86 (anti-CD86-PerCP-cy5.5, BioLegend, catalog number: 305430) were used for labeling. Cell evaluation was performed using the MACSQuant flow cytometer (Miltenyi Biotec, Auburn, CA, USA), followed by analysis of the results using FlowJo v10.5.3 software (BD Biosciences).

### Autologous CD3 + T cell isolation process

Autologous CD3 + T cells were isolated from PBMCs of the same donor used for DC culture using Magnetic-Activated Cell Sorting (MACS) and the Human Pan T Cell Isolation Kit (Miltenyi Biotec, part number: 130–096-535). Briefly, following the separation of PBMCs, the cell suspension was subjected to centrifugation at 300 × g for a period of 10 min. The supernatant was removed, and for every 1 × 10^7^ cells, the mixture contained 10 µL of the pan-T cell biotin antibody mix and 40 µL of MACS buffer was introduced. After incubation at 2–8°C for 5 min, the addition involved introducing 30 µL of MACS buffer along with 20 µL of the pan-T cell microbeads mix. The cells underwent a cleansing process using MACS buffer, were then placed in cold conditions between 2–8°C for 10 min, and finally suspended in 500 µL of MACS buffer. The cell suspension was loaded onto a MACS column and placed in the magnetic field of a MACS separator. Cells flowing through the column without labeling were indicative of the CD3 + T cells selected negatively in the process.

### Assessment of CD3 + T lymphocyte proliferation using CFSE labeling

CFSE (Carboxyfluorescein Succinimidyl Ester) was employed to tag the isolated CD3 + T cells, adhering to the instructions outlined by the manufacturer. In a brief outline, the separated T cells were resuspended in PBS and incubated with 5 µM CFSE at 25 °C, under light-protected conditions for 5 min. The reaction was terminated through supplementing RPMI-1640 medium with 20% FBS. After washing, the collected cells were transferred to pre-warmed culture medium. To evaluate the ability of mature DCs and SPACA6P-AS silenced mDCs to promote the proliferation of autologous T cells, co-culture experiments of DCs and T cells were conducted. In a V-bottom 96-well plate, control group mDCs and silenced SPACA6P-AS mDCs were co-cultured with CFSE-labeled autologous CD3 + T lymphocytes at ratios of 1:5 and 1:10. The positive control group included T cells activated with 5% phytohemagglutinin (Sigma Chemical Co., part number: L4144-10MG), while the unstimulated group consisted of T cells co-cultured with iDCs. Upon completion of a four-day incubation period in a light-shielded environment, the expansion of T cells tagged with CFSE was evaluated via flow cytometry analysis. The control group was made up of unlabeled CD3 + T lymphocytes.

### Immunohistochemical staining

For immunohistochemical staining, cells were first fixed with 4% formaldehyde solution (Servicebio, G1101) and dehydrated and cleared with xylene (Fisher Scientific, X3-1L) and graded ethanol concentrations (Fisher Scientific, BP2818-4). The tissue samples were subsequently encased in paraffin and cut into sections with a thickness of 4 µm. The segments were subjected to a heating process at 58 °C for a duration of 30 min to remove paraffin, subsequent to antigen retrieval in citrate buffer (pH 6.0, Servicebio, CL-K943). The endogenous peroxidase activity was suppressed by treatment with 3% hydrogen peroxide (Sigma-Aldrich, H1009), and nonspecific binding was blocked with 5% BSA (Sigma-Aldrich, A9647). The specimens were subsequently subjected to an overnight incubation in the presence of the Ki-67 antibody (Abcam, ab15580). The following day, secondary antibodies and DAB chromogen (DAKO, K5007) were applied, and nuclei were counterstained with hematoxylin. Slides were then mounted.

### Cytokine assay

Recently harvested CD3 + T cells were cultured together with control mDCs and SPACA6P-AS-silenced mDCs at a 1:5 ratio in 24-well plates to study the capabilities of control and SPACA6P-AS-silenced mDCs to stimulate cytokine secretion when autologous T cells are around. Post a 48-h DC stimulation, supernatants were harvested from the co-culture, and the concentrations of IFN-γ, TGF-β, and IL-4 were assessed using ELISA kits from R&D systems (Minneapolis, Minnesota, USA, DY285-05, DY240-05, and DY204-05). Levels of IL-12 and IL-10 in the supernatants of control and SPACA6P-AS-silenced mDCs were also assess through ELISA kits (R&D Systems, Minneapolis, Minnesota, USA, DY1270-05).

### Statistical analysis

The statistical analysis predominantly employed R software (version 4.2.0). DEGs was identified using the DESeq2 R package, with a threshold set at |log2 FC|> 1.5, and significance determined by Benjamini–Hochberg method with adjusted p-value < 0.05. For survival analysis, the The Cox proportional hazards regression model was performed to evaluate the relationship between lncRNA SPACA6P-AS expression and patient survival rates, calculating the survival rates at 1, 3, and 5 years. The estimation of survival curves was carried out utilizing the Kaplan–Meier (KM) approach, while variation in survival outcomes between different cohorts was assessed using log-rank tests. GO and KEGG pathway enrichment analyses, as well as GSEA, were employed via the clusterProfiler package and GSEA software, respectively. The GSEA analysis was based on 1000 permutations to obtain normalized enrichment scores (NES), with significance set at NOM *p* < 0.05. Immune infiltration analysis using ssGSEA was carried out with the GSVA R package, and Spearman rank correlation coefficients were used for correlation analysis. Two-tailed statistical tests were utilized with a significance threshold set at *p* < 0.05. In situations involving multiple comparisons, adjustments of p-values were carried out using the Benjamini–Hochberg technique. A minimum of three trials were performed for each test, with outcomes shown as mean ± standard deviation (SD). Group contrasts were evaluated utilizing Analysis of Variance (ANOVA) accompanied by post-hoc Tukey HSD analysis when required (GraphPad Prism, GraphPad Software, Version 8.0). Correlation analysis was carried out using Spearman or Pearson correlation coefficients (IBM SPSS Statistics, IBM, Version 25), with significance considered at *p* < 0.05.

## Results

### Significant increase in LncRNA SPACA6P-AS expression in BC patients in TCGA

**Fig. 1:**
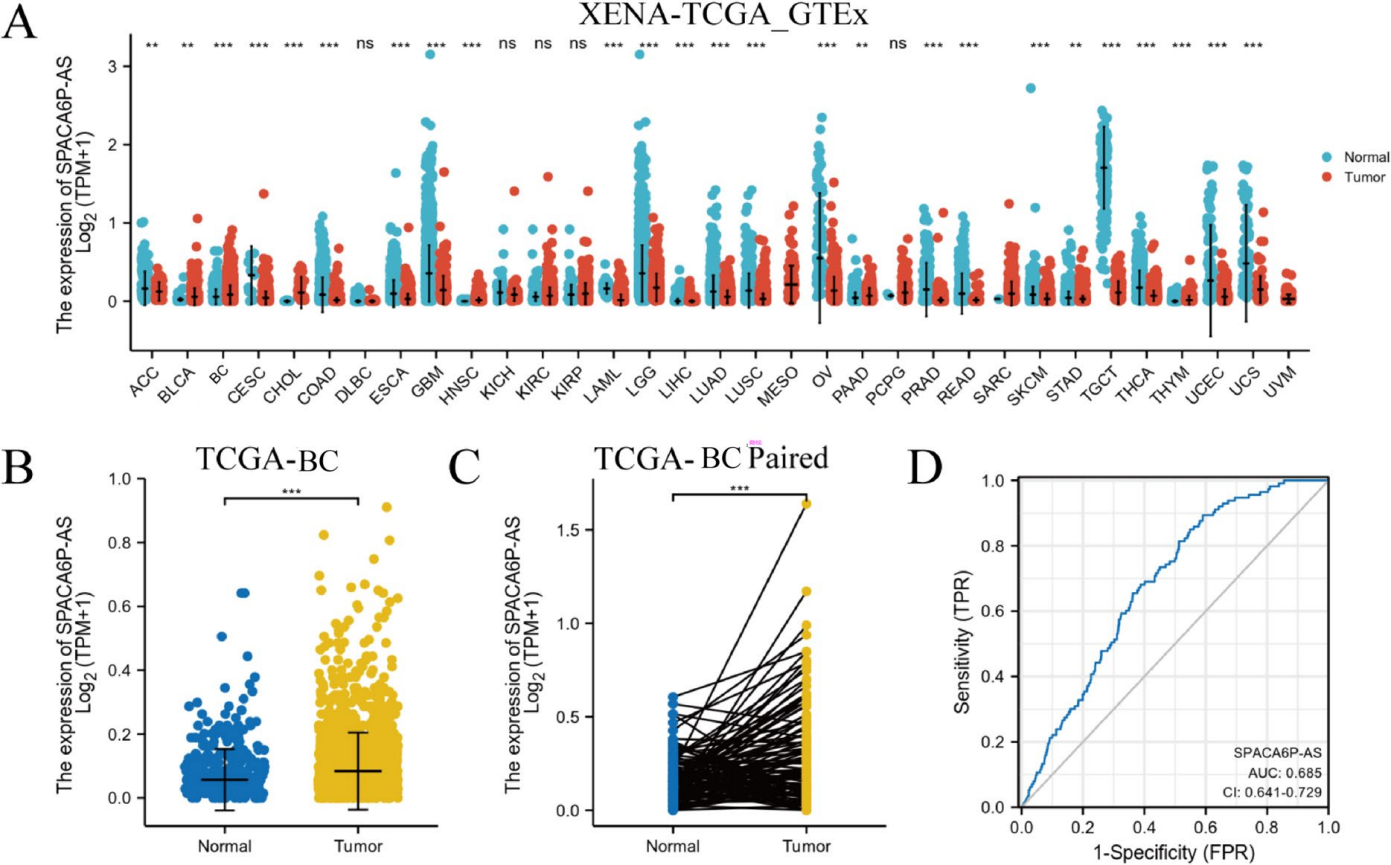
Expression of lncRNA SPACA6P-AS in various cancers and its potential diagnostic value in BC. *Note*: **A** Comparative analysis of lncRNA SPACA6P-AS expression levels in tumor (*N* = 1083) and normal (*N* = 292) tissue samples of various cancer types from The Cancer Genome Atlas (TCGA; https://portal.gdc.cancer.gov/) and the Genotype-Tissue Expression (GTEx; https://www.gtexportal.org/home/index.html) databases reveals significant upregulation in multiple cancers. **B** Contrasting the expression levels of lncRNA SPACA6P-AS in tumor tissues (*N* = 1083) of BC patients from the TCGA-BC project with normal breast tissues (*N* = 292) indicates a marked increase in expression in BC tissue. **C** Validation through paired sample analysis (*N* = 113) confirms the significant upregulation of lncRNA SPACA6P-AS in BC tissue, further supporting its potential as a biomarker for BC. **D** Analysis of the receiver operating characteristic (ROC) curve assesses the predictive value of lncRNA SPACA6P-AS in discriminating BC tissue from normal tissue, yielding an AUC value of 0.685. Statistical significance levels are denoted by asterisks: *p* < 0.05, *p* < 0.01, **p < 0.001, and "ns" indicates nonsignificance

Our study extensively analyzed tumor and standard tissue samples from the TCGA and GTEx databases, focusing on the expression differences of lncRNA SPACA6P-AS across various cancers (Fig. 1A). A careful comparison of the data revealed significant upregulation of lncRNA SPACA6P-AS in multiple cancers, including bladder cancer (Ascione et al. 2023), BC (Nolan et al. 2023), head and neck squamous cell carcinoma (Johnson et al. 2020), and pancreatic cancer (Sherman and Beatty 2023), with a particularly notable increase in BC. Detailed clinical and gene expression data of 1083 BC patients were obtained through download from the TCGA database for an in-depth analysis (Table 1). Results showed a notable rise in the lncRNA SPACA6P-AS expression within BC tissues in contrast to the usual mammary tissues (Fig. 1B), further validated by paired sample analysis (*p* < 0.001) (Fig. 1C). The accuracy of lncRNA SPACA6P-AS in distinguishing BC tissue from normal tissue was evaluated utilizing receiver operating characteristic (ROC) curve analysis. The results indicated an area under the curve (AUC) of 0.685, surpassing random guessing and demonstrating predictive value (Fig. 1D). These comprehensive findings suggest that the expression of lncRNA SPACA6P-AS is notably upregulated in BC tissue relative to normal tissue, warranting further investigation in clinical and biological research.

**Table 1:** Clinical information on 1083 breast cancer patients

Characteristic	Low expression of SPACA6P-AS	High expression of SPACA6P-AS	*p*
*n*	541	542	
T stage, n (%)			0.718
T1	141 (13.1%)	136 (12.6%)	
T2	307 (28.4%)	322 (29.8%)	
T3	75 (6.9%)	64 (5.9%)	
T4	17 (1.6%)	18 (1.7%)	
N stage, n (%)			0.214
N0	260 (24.4%)	254 (23.9%)	
N1	166 (15.6%)	192 (18%)	
N2	65 (6.1%)	51 (4.8%)	
N3	42 (3.9%)	34 (3.2%)	
M stage, n (%)			0.242
M0	444 (48.2%)	458 (49.7%)	
M1	13 (1.4%)	7 (0.8%)	
Pathologic stage, n (%)			0.249
Stage I	93 (8.8%)	88 (8.3%)	
Stage II	296 (27.9%)	323 (30.5%)	
Stage III	128 (12.1%)	114 (10.8%)	
Stage IV	12 (1.1%)	6 (0.6%)	
Race, n (%)			0.004
Asian	37 (3.7%)	23 (2.3%)	
Black or African American	73 (7.3%)	108 (10.9%)	
White	389 (39.1%)	364 (36.6%)	
Age, n (%)			0.649
< = 60	296 (27.3%)	305 (28.2%)	
> 60	245 (22.6%)	237 (21.9%)	
Histological type, n (%)			0.234
Infiltrating Ductal Carcinoma	372 (38.1%)	400 (40.9%)	
Infiltrating Lobular Carcinoma	109 (11.2%)	96 (9.8%)	
PR status, n (%)			0.303
Negative	182 (17.6%)	160 (15.5%)	
Indeterminate	3 (0.3%)	1 (0.1%)	
Positive	339 (32.8%)	349 (33.8%)	
ER status, n (%)			0.651
Negative	129 (12.5%)	111 (10.7%)	
Indeterminate	1 (0.1%)	1 (0.1%)	
Positive	394 (38.1%)	399 (38.6%)	
HER2 status, n (%)			0.189
Negative	277 (38.1%)	281 (38.7%)	
Indeterminate	5 (0.7%)	7 (1%)	
Positive	90 (12.4%)	67 (9.2%)	
PAM50, n (%)			< 0.001
Normal	28 (2.6%)	12 (1.1%)	
LumA	287 (26.5%)	275 (25.4%)	
LumB	78 (7.2%)	126 (11.6%)	
Her2	52 (4.8%)	30 (2.8%)	
Basal	96 (8.9%)	99 (9.1%)	
Menopause status, n (%)			0.165
Pre	114 (11.7%)	115 (11.8%)	
Peri	26 (2.7%)	14 (1.4%)	
Post	349 (35.9%)	354 (36.4%)	
Anatomic neoplasm subdivisions, n (%)			0.412
Left	274 (25.3%)	289 (26.7%)	
Right	267 (24.7%)	253 (23.4%)	
radiation_therapy, n (%)			0.056
No	230 (23.3%)	204 (20.7%)	
Yes	258 (26.1%)	295 (29.9%)	
Age, median (IQR)	58 (49, 68)	58 (48, 66.75)	0.230

This table presents a comprehensive analysis comparing the clinical and pathological characteristics of breast cancer patients with low versus high expression of lncRNA SPACA6P-AS. The data is segmented into various categories including T stage, N stage, M stage, pathologic stage, race, age, histological type, PR status, ER status, HER2 status, PAM50 subtype, menopause status, anatomic neoplasm subdivisions, and radiation therapy status. The number and percentage of patients in each category are displayed for both low and high expression groups. The p-values, calculated to assess the statistical significance of differences between the two groups, are also provided for each characteristic. The median age and interquartile range (IQR) are included at the bottom of the table. This tabular representation aids in understanding the potential correlations between SPACA6P-AS expression levels and various clinical attributes in breast cancer patients

### LncRNA SPACA6P-AS expression correlation with clinicopathological features in BC patients

**Fig. 2:**
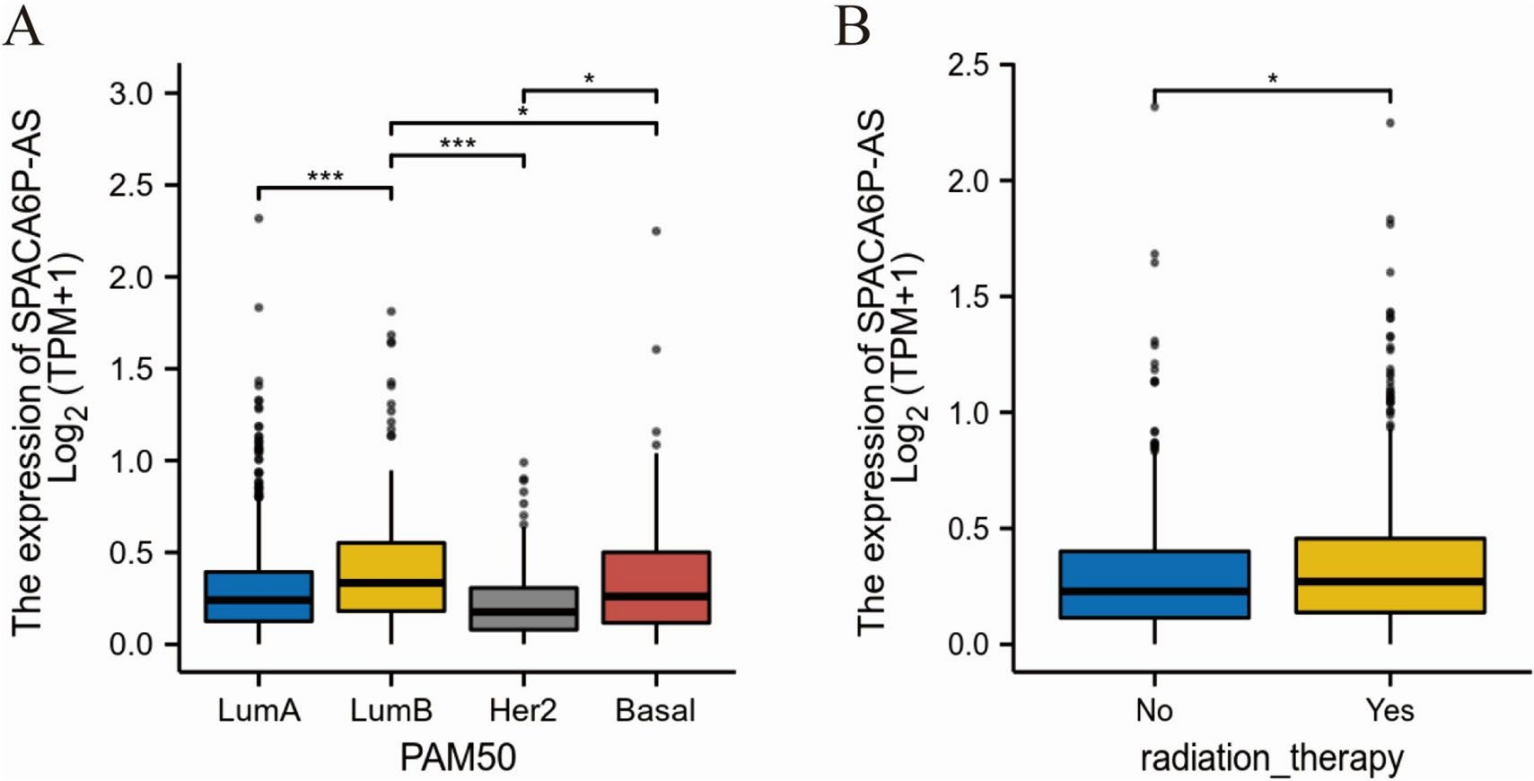
Relationship between lncRNA SPACA6P-AS expression and PAM50 subtypes and radiation therapy history in BC patients. *Note*: **A** Box plots showing lncRNA SPACA6P-AS expression levels across different PAM50 subtypes, including LumA, LumB, Her2, and Basal. **B** Box plots of lncRNA SPACA6P-AS expression levels in patients who received radiation therapy versus those who did not. In the box plots, the box represents the interquartile range, the horizontal line represents the median, whiskers show the data variability, and outliers are marked as individual points. Asterisks indicate levels of statistical significance: *p < 0.05, ***p < 0.001

**Fig. 3:**
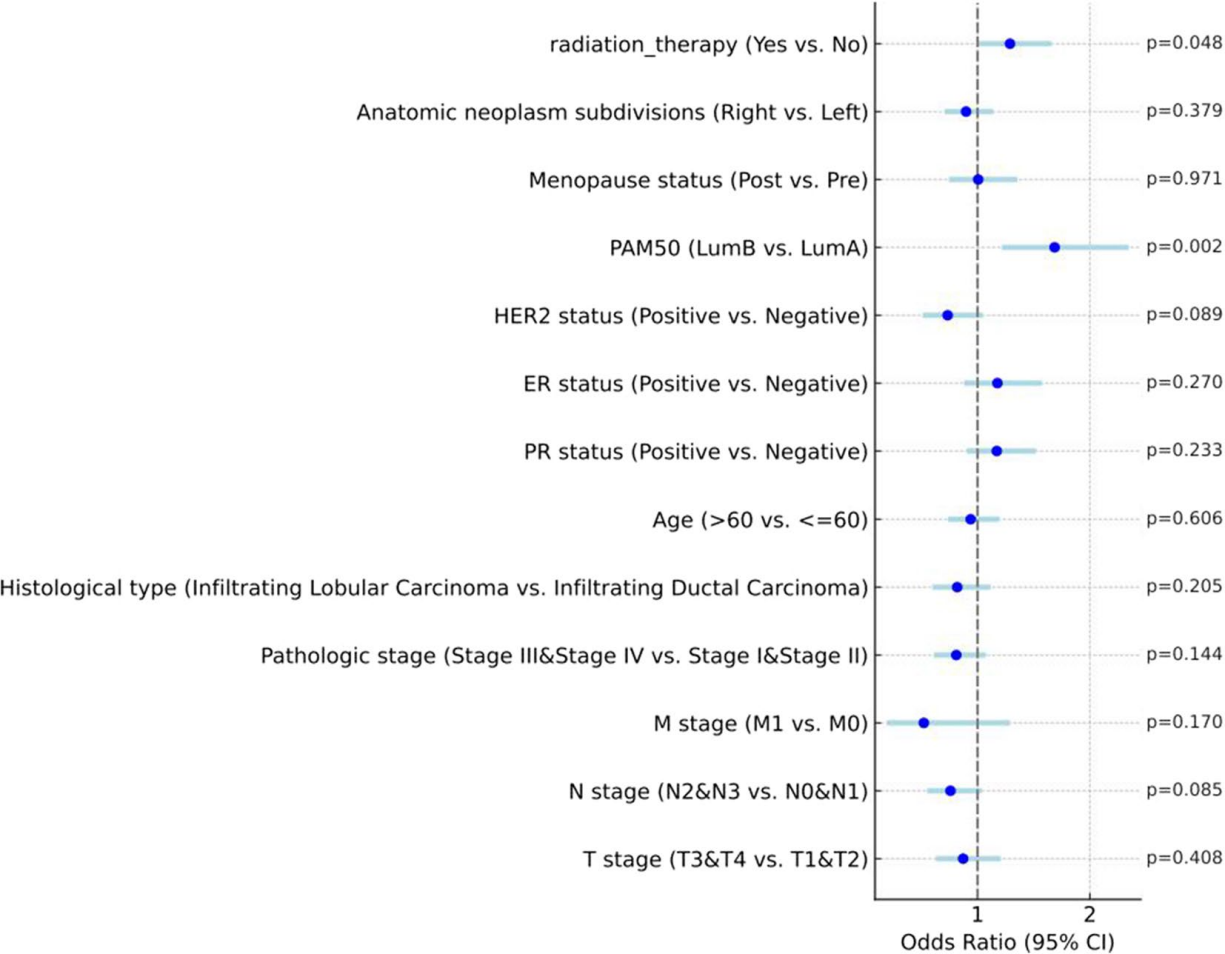
ORs forest plot of various clinical and pathological features in BC patients. *Note*: This forest plot displays the odds ratios (ORs) and their 95% confidence intervals (CIs) for various clinical and pathological features in BC patients. The central points represent ORs, while the horizontal lines indicate 95% CIs. An OR greater than 1 indicates an increased risk or likelihood associated with that feature, while an OR less than 1 indicates a reduced risk. P-values next to each line signify the statistical significance of these associations. The vertical grey dashed line at OR = 1 is a reference point for no effect

In our study, BC patients were grouped taking into consideration the median expression of long non-coding RNA SPACA6P-AS into a high-expression group (*N* = 542) and a low-expression group (*N* = 541). By analyzing the correlation between the clinical-pathological characteristics of these two patient groups and the expression levels of SPACA6P-AS, we found a significant association between increased SPACA6P-AS expression and the PAM50 subtypes of BC as well as a history of receiving radiotherapy (Table 1, Fig. 2). Expressly, univariate logistic regression analysis indicated a significant correlation of high SPACA6P-AS expression with LumB subtype compared to LumA (odds ratio (OR) = 1.686, *p* = 0.02) and individuals who underwent radiation therapy in contrast to those individuals who did not have this therapy (OR = 1.289, *p* = 0.048) (Table 2, Fig. 3).

**Table 2:** Correlation analysis between clinical pathological characteristics and lncRNA SPACA6P-AS expression level in breast cancer patients

Characteristics	Total(N)	Odds Ratio(OR)	*P* value
T stage (T3&T4 vs. T1&T2)	1,080	0.872 (0.629–1.206)	0.408
N stage (N2&N3 vs. N0&N1)	1,064	0.759 (0.553–1.038)	0.085
M stage (M1 vs. M0)	922	0.522 (0.195–1.287)	0.170
Pathologic stage (Stage III&Stage IV vs. Stage I&Stage II)	1,060	0.811 (0.612–1.074)	0.144
Histological type (Infiltrating Lobular Carcinoma vs. Infiltrating Ductal Carcinoma)	977	0.819 (0.601–1.115)	0.205
Age (> 60 vs. < = 60)	1,083	0.939 (0.739–1.193)	0.606
PR status (Positive vs. Negative)	1,030	1.171 (0.903–1.519)	0.233
ER status (Positive vs. Negative)	1,033	1.177 (0.881–1.573)	0.270
HER2 status (Positive vs. Negative)	715	0.734 (0.512–1.047)	0.089
PAM50 (LumB vs. LumA)	766	1.686 (1.218–2.345)	0.002
Menopause status (Post vs. Pre)	932	1.006 (0.746–1.355)	0.971
Anatomic neoplasm subdivisions (Right vs. Left)	1,083	0.898 (0.708–1.140)	0.379
radiation_therapy (Yes vs. No)	987	1.289 (1.002–1.659)	0.048

This table presents the correlation between the expression level of lncRNA SPACA6P-AS and various clinical pathological characteristics in breast cancer patients, based on data from 1,083 patients. The clinical pathological features analyzed include T stage, N stage, M stage, pathologic stage, histological type, age, progesterone receptor (PR) status, estrogen receptor (ER) status, HER2 status, PAM50 subtype, menopausal status, and anatomic neoplasm subdivisions (right vs. left). The analysis for each characteristic includes the total number of samples (Total(N)), Odds Ratio (OR) along with its 95% confidence interval, and the *P* value. An OR value greater than 1 indicates an increased risk associated with high SPACA6P-AS expression, while a value less than 1 indicates a decreased risk. A *P* value less than 0.05 is considered statistically significant. Notably, the PAM50 subtype (LumB vs. LumA) and receiving radiation therapy (Yes vs. No) are significantly associated with high SPACA6P-AS expression

Additionally, in the univariate analysis of overall survival (OS) prognostic factors, we found a significant correlation between high expression of the long non-coding RNA SPACA6P-AS and adverse prognosis (*p* = 0.004, hazard ratio [HR] = 1.616, 95% confidence interval [1.169–2.235]). Similarly, high TNM staging, advanced pathological stage, older age, and specific PAM50 subtypes were significantly associated with poor OS. Specifically, higher T stage (*p* = 0.012, HR = 1.608, 95% CI [1.110–2.329]), N stage (*p* < 0.001, HR = 2.163, 95% CI [1.472–3.180]), M stage (*p* < 0.001, HR = 4.254, 95% CI [2.468–7.334]), higher pathological stage (*p* < 0.001, HR = 2.391, 95% CI [1.703–3.355]), and older age (*p* < 0.001, HR = 2.020, 95% CI [1.465–2.784]) were all correlated with poor OS (Table 3). Within PAM50 subtypes, LumA compared to Her2 (*p* < 0.003, HR = 2.405, 95% CI [1.325–3.859]) also showed a significant adverse prognosis.

**Table 3:** Impact of clinical pathological characteristics and lncRNA SPACA6P-AS expression on breast cancer prognosis: univariate and multivariate analysis

Characteristics	Total(N)	Univariate analysis		Multivariate analysis	
		Hazard ratio (95% CI)	*P* value	Hazard ratio (95% CI)	*P* value
T stage	1079				
T1&T2	905	Reference			
T3&T4	174	1.608 (1.110–2.329)	0.012	2.796 (1.184–6.603)	0.019
N stage	1063				
N0&N1	871	Reference			
N2&N3	192	2.163 (1.472–3.180)	< 0.001	5.502 (1.340–22.591)	0.018
M stage	922				
M0	902	Reference			
M1	20	4.254 (2.468–7.334)	< 0.001	2.414 (0.565–10.314)	0.234
Pathologic stage	1059				
Stage I&Stage II	799	Reference			
Stage III&Stage IV	260	2.391 (1.703–3.355)	< 0.001	1.022 (0.238–4.398)	0.976
Race	993				
Asian	60	Reference			
Black or African American	180	1.525 (0.463–5.024)	0.488		
White	753	1.325 (0.420–4.186)	0.631		
Age	1082				
< = 60	601	Reference			
> 60	481	2.020 (1.465–2.784)	< 0.001	2.735 (1.288–5.809)	0.009
Histological type	977				
Infiltrating Ductal Carcinoma	772	Reference			
Infiltrating Lobular Carcinoma	205	0.827 (0.526–1.299)	0.410		
PR status	1029				
Negative	342	Reference			
Positive	687	0.732 (0.523–1.024)	0.068	0.804 (0.279–2.312)	0.685
ER status	1032				
Negative	240	Reference			
Positive	792	0.712 (0.495–1.023)	0.066	0.258 (0.056–1.185)	0.082
HER2 status	715				
Negative	558	Reference			
Positive	157	1.593 (0.973–2.609)	0.064	0.912 (0.377–2.209)	0.839
PAM50	1042				
LumA	561	Reference			
LumB	204	1.663 (1.088–2.541)	0.019	1.248 (0.539–2.888)	0.604
Her2	82	2.261 (1.325–3.859)	0.003	0.486 (0.086–2.741)	0.414
Basal	195	1.285 (0.833–1.981)	0.257	0.610 (0.112–3.336)	0.569
Menopause status	931				
Pre	229	Reference			
Post	702	2.165 (1.302–3.600)	0.003	3.969 (1.238–12.722)	0.020
Anatomic neoplasm subdivisions	1082				
Left	563	Reference			
Right	519	0.766 (0.554–1.057)	0.105		
radiation_therapy	986				
No	434	Reference			
Yes	552	0.576 (0.394–0.841)	0.004	0.424 (0.209–0.860)	0.017
SPACA6P-AS	1082				
Low	540	Reference			
High	542	1.616 (1.169–2.235)	0.004	2.189 (1.091–4.395)	0.028

This table provides an extensive analysis of the influence of various clinical pathological characteristics and the expression of lncRNA SPACA6P-AS on the prognosis of breast cancer patients. The data encompasses a total of 1,082 patients, assessing factors such as T stage, N stage, M stage, pathologic stage, race, age, histological type, PR status, ER status, HER2 status, PAM50 subtype, menopause status, anatomic neoplasm subdivisions, radiation therapy, and SPACA6P-AS expression level. For each characteristic, the table presents the Hazard Ratios (HR) with 95% Confidence Intervals (CI) and *P* values, obtained from both univariate and multivariate analysis. A HR greater than 1 indicates an increased risk, while a HR less than 1 suggests a decreased risk. Significant *P* values (*P* < 0.05) denote statistically relevant correlations. This table aids in understanding the complex interplay of these factors in predicting breast cancer prognosis

Further multivariate Cox regression analysis identified SPACA6P-AS expression level (*p* = 0.028, HR = 2.189, 95% CI [1.091–4.395]) as an independent predictor of overall survival in BC. Additionally, T stage (*p* = 0.019, HR = 2.796, 95% CI [1.184–6.603]), N stage (*p* < 0.018, HR = 5.502, 95% CI [1.340–22.591]), age (*p* = 0.009, HR = 2.735, 95% CI [1.288–5.809]), menopausal status (*p* = 0.020, HR = 3.969, 95% CI [1.238–12.722]), and radiation therapy (*p* = 0.017, HR = 0.424, 95% CI [0.209–0.860]) were also identified as significant factors affecting overall survival in BC patients (Table 3). These results underscore the significant role of SPACA6P-AS in the prognostic assessment of BC, highlighting its potential as a therapeutic target.

### Correlation between LncRNA SPACA6P-AS expression and survival in BC patients

**Fig. 4:**
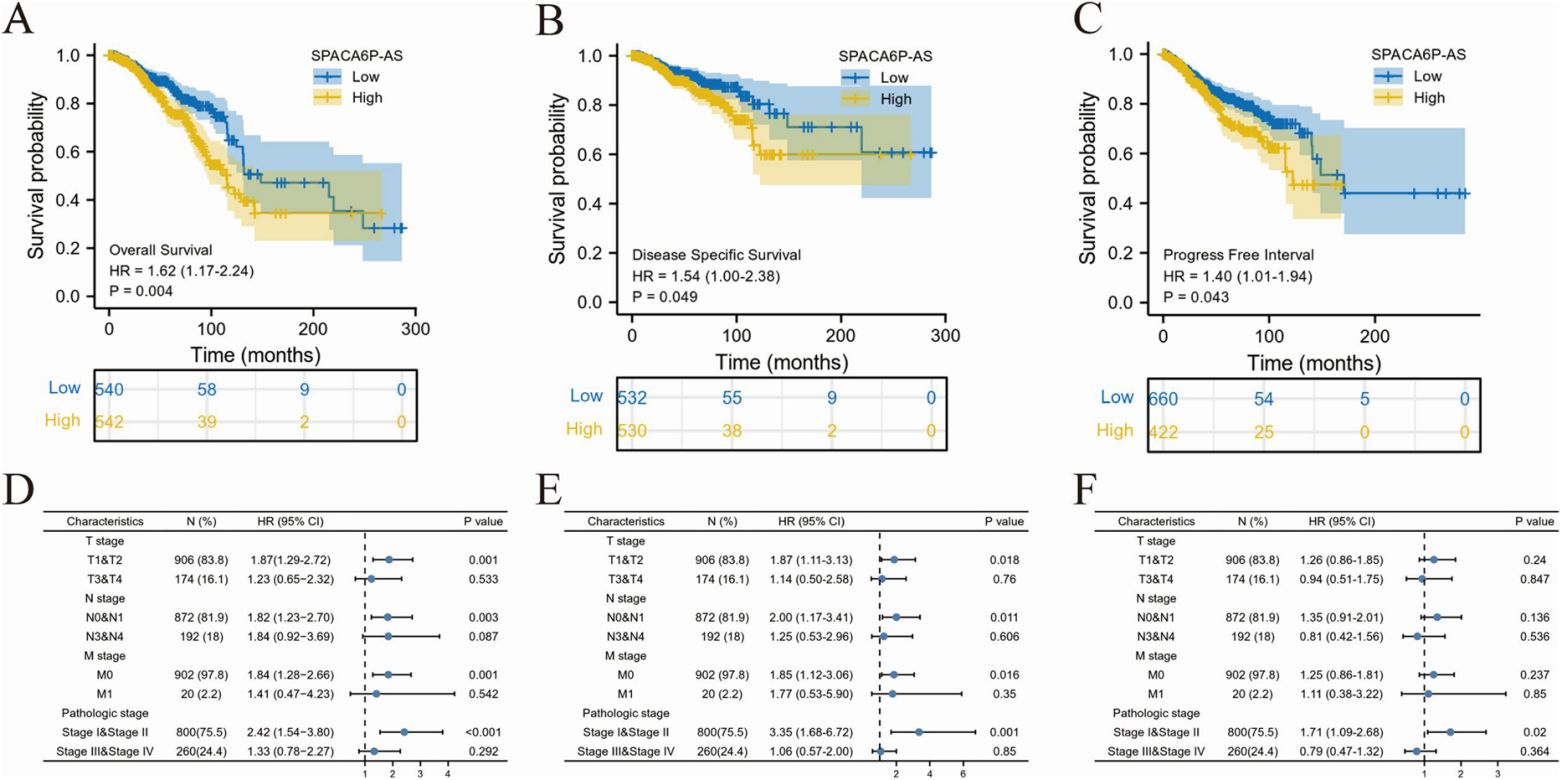
Survival analysis of lncRNA SPACA6P-AS expression in BC patients. *Note*: **A**-**C**: Kaplan–Meier survival curves demonstrating the correlation of lncRNA SPACA6P-AS expression with survival prognosis in BC patients. **A** OS analysis shows significantly longer survival in the high SPACA6P-AS expression group compared to the low expression group. **B** DSS analysis reveals higher survival rates in the high SPACA6P-AS expression group. **C** PFI analysis indicates shorter disease-free periods in the high SPACA6P-AS expression group. **D**-**F** The impact of SPACA6P-AS in TNM staging on OS of BC patients. **E** The impact of SPACA6P-AS in TNM staging on DSS of BC patients. **F** The impact of SPACA6P-AS in TNM staging on PFS of BC patients

In our study utilizing data from TCGA database, KM survival curve analysis revealed that individuals with high SPACA6P-AS expression exhibited significantly prolonged overall survival (OS) when contrasted with the low-expression group, with a HR of 1.62 (95% confidence interval 1.17–2.24) indicating a statistically significant difference (*p* = 0.004) (Fig. 4A-C). This suggests that elevated levels of lncRNA SPACA6P-AS could serve as a favorable prognostic indicator for BC patients. Furthermore, in the analysis of Progression-Free Interval (PFI), we observed that BC patients with high SPACA6P-AS expression had a shorter PFI, with a HR of 1.40 (95% CI 1.01–1.94) and a p-value of 0.043 (Fig. 4D-F), indicating a higher risk of recurrence or disease progression for these patients. Moreover, in the analysis of Disease-Specific Survival (DSS), patients in the low SPACA6P-AS expression group exhibited higher DSS compared to the high-expression group, with a HR of 1.54 (95% CI 1.00–2.38) and a p-value of 0.049.

**Fig. 5:**
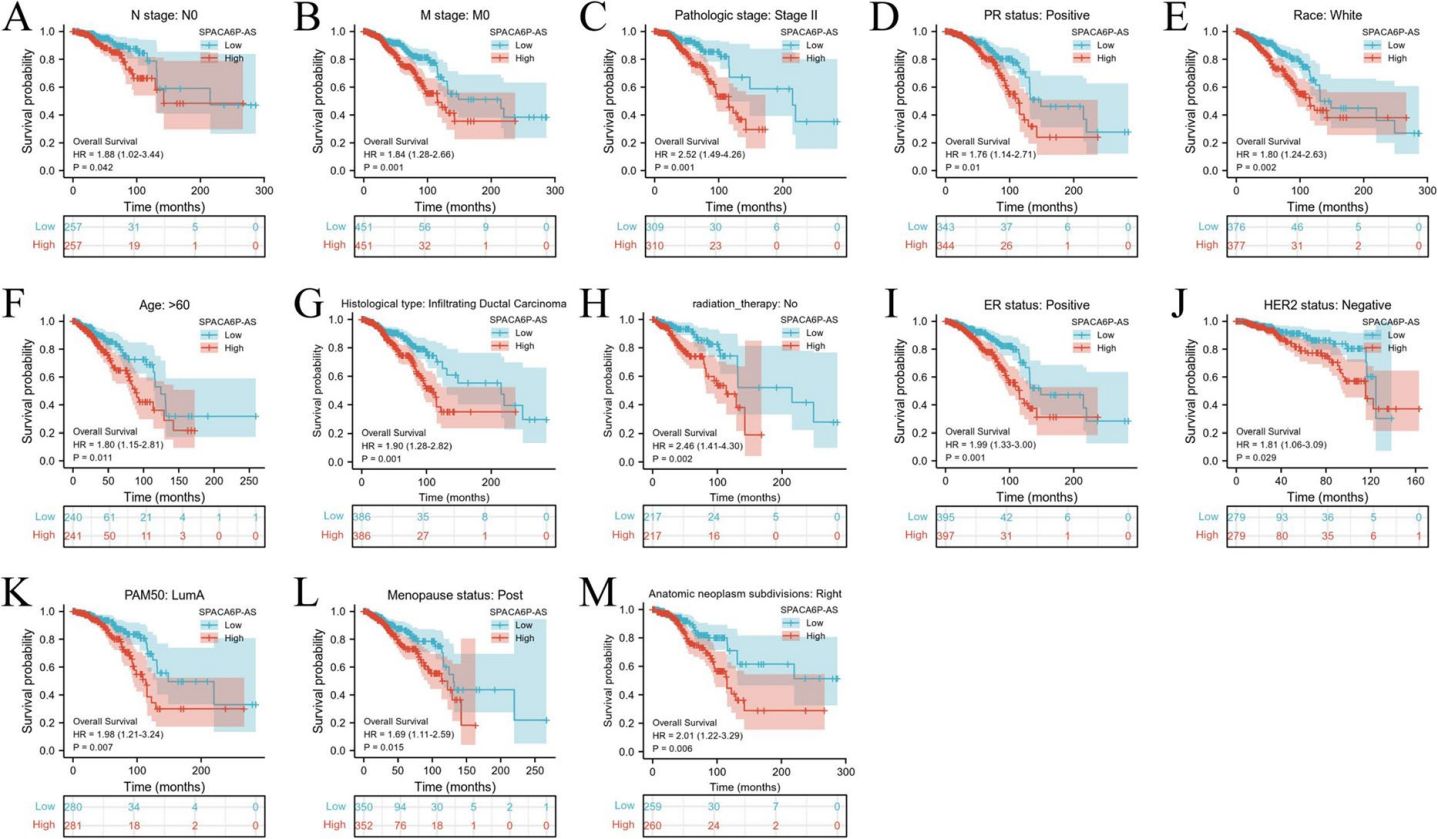
Survival prognosis analysis of lncRNA SPACA6P-AS in BC patients with different clinicopathological features. *Note*: **A**-**M**: Multiple Kaplan–Meier survival curves displaying the correlation of lncRNA SPACA6P-AS expression levels with survival prognosis according to different clinicopathological features in BC patients. **A** Survival curve for N stage-N0 group. **B** M stage-M0 group. **C** Pathological stage II group. **D** PR positive status group. **E** Caucasian race group. **F** Age over 60 group. **G** Invasive ductal carcinoma group. **H** No radiation therapy group. **I** ER positive status group. **J** HER2 negative status group. **K** PAM50-LumA subtype group. **L** Postmenopausal status group. **M** Anatomical tumor subdivision right side group

Using a multivariate risk model, we further evaluated the impact of SPACA6P-AS in TNM staging on overall survival, PFS, and DSS of BC patients. The analysis demonstrated the significant prognostic value of SPACA6P-AS expression levels across various clinicopathological feature subgroups, including N stage-N0, M stage-M0, pathological stage II, progesterone receptor (PR) positive, Caucasian race, age over 60, invasive ductal carcinoma, no radiation therapy, Estrogen Receptor (ER) positive, Human Epidermal Growth Factor Receptor 2 (HER2) negative, PAM50-LumA subtype, postmenopausal status, and anatomical tumor subdivision (Fig. 5). In these subgroups, a decrease in lncRNA SPACA6P-AS levels was closely related to poor prognosis, emphasizing the importance of SPACA6P-AS in prognosis assessment and its potential clinical application value in BC.

### Construction and validation of a predictive model based on LncRNA SPACA6P-AS

**Fig. 6:**
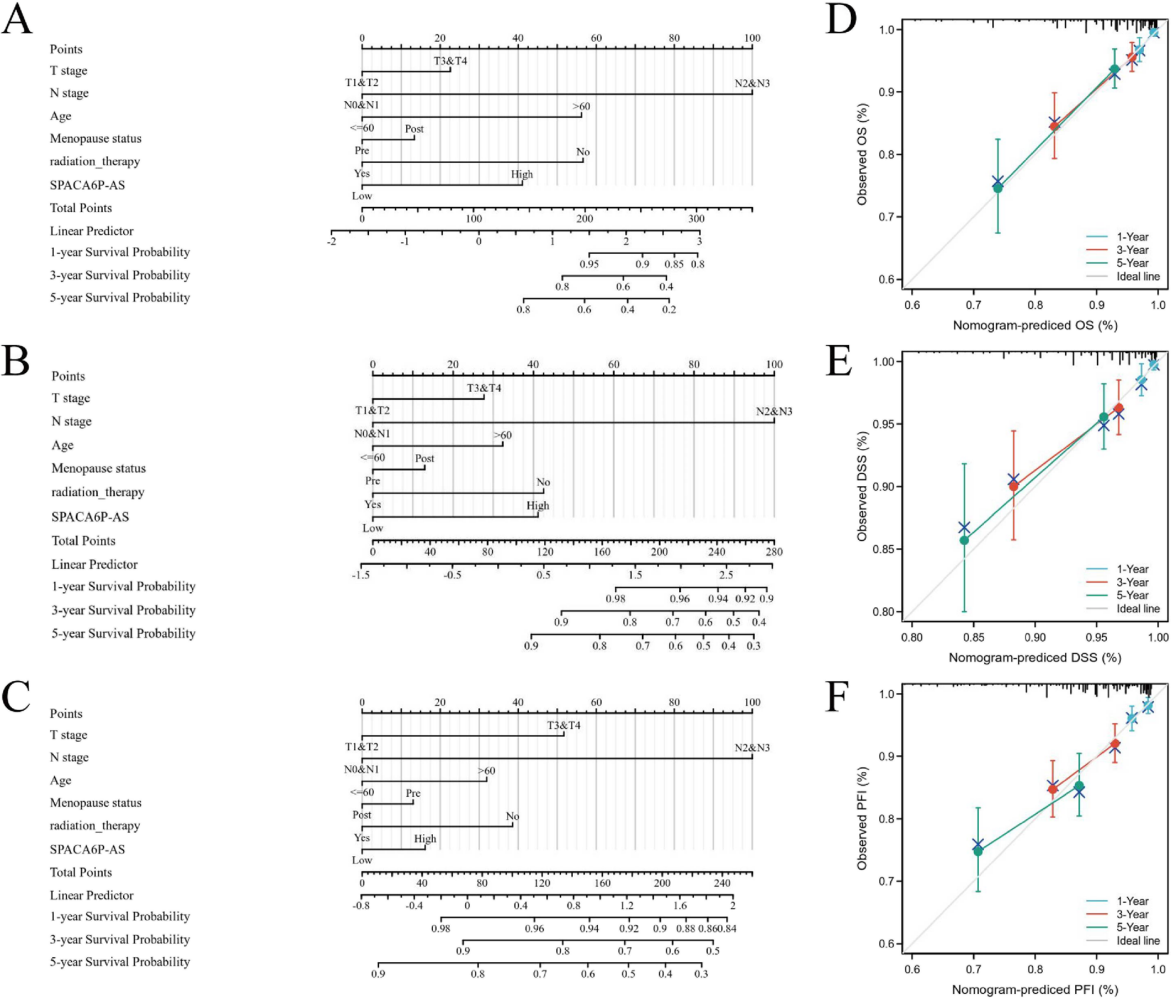
Prognostic nomogram and calibration analysis for BC based on lncRNA SPACA6P-AS and clinical factors. *Note*: **A**-**C**: Prognostic nomograms for BC patients, incorporating T stage, N stage, age, menopausal status, radiation therapy, and SPACA6P-AS expression level. Each subfigure's bar graph represents the corresponding prognostic probability, including 1-year, 3-year, and 5-year OS, DSS, and PFI. **A** OS nomogram. **B** DSS nomogram. **C** PFI nomogram. **D**-**F**: Calibration curves evaluating the consistency of predicted and observed survival probabilities. Each curve matches prognostic predictions to actual outcomes at different time points. **D** OS calibration curve. **E** DSS calibration curve. **F** PFI calibration curve. In calibration curves, each point represents the relationship between the nomogram's predicted survival probabilities and actual survival data, with the ideal line indicating perfect prediction. The C-index shows the high accuracy of the model in predicting BC prognosis. The 95% CIs obtained from 1,000 bootstrap resamples further confirm the reliability of the predictions

To quantify the prognosis of BC patients, we developed a nomogram that integrates SPACA6P-AS with independent clinical risk factors, including T stage, N stage, age, menopausal status, and radiation therapy. The overall scores of the nomograms for OS, DSS, and PFI were relatively high, indicating a poorer prognosis (Fig. 6). The results demonstrated a high concordance between the model's predictions and actual observations, suggesting excellent model fit. The model's C-index was 0.749, indicating high discriminative ability. The 95% CI for OS prediction, determined through 1,000 bootstrap resamples, was 0.719–0.779. Similarly, the C-index for DSS was 0.749 (95% CI [0.709–0.789]), and for PFI, it was 0.668 (CI [0.636–0.699]). The calibration plots' bias-corrected lines closely approached the ideal curve (Keynesian crossover), further emphasizing the strong correlation between predicted and observed values. Overall, these results demonstrate that our SPACA6P-AS-based predictive model is statistically significant and clinically practical, effectively forecasting both long-term and short-term survival in BC patients and providing a crucial decision-making tool for clinicians.

### Potential mechanisms of LncRNA SPACA6P-AS in regulating BC progression

**Fig. 7:**
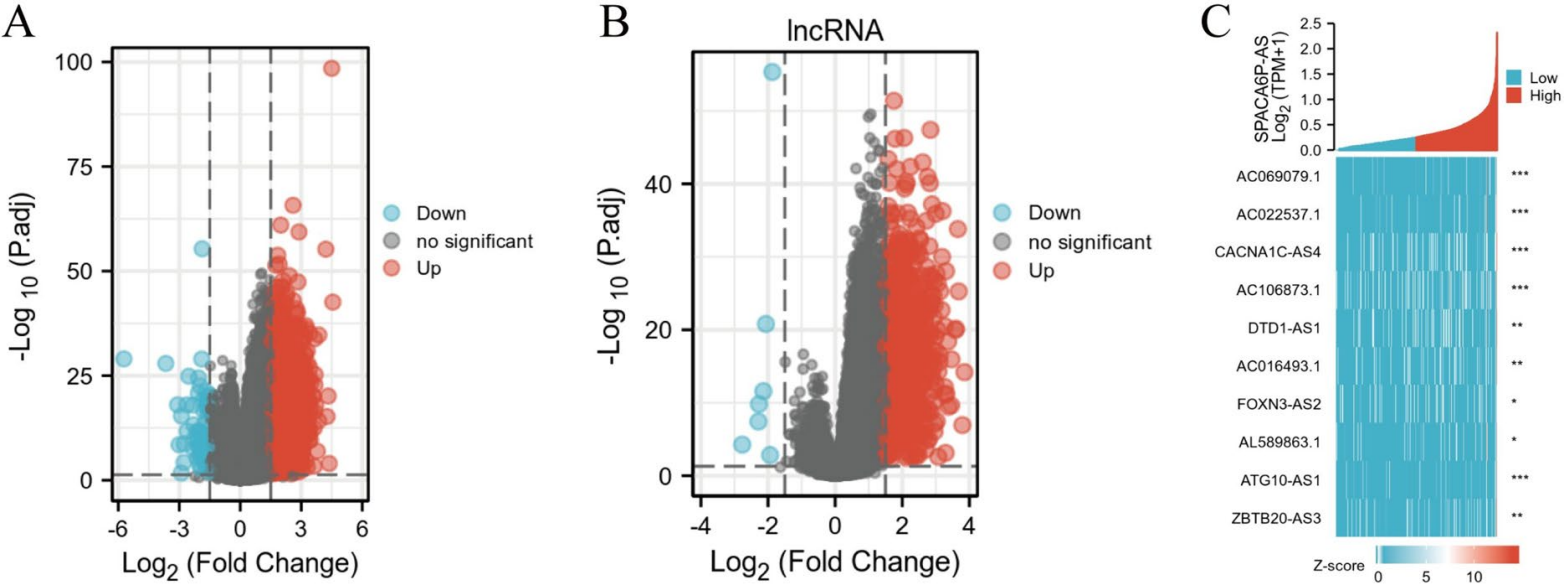
Differential expression analysis revealing lncRNA SPACA6P-AS associated genes and lncRNAs in BC. *Note*: **A** Volcano plot of whole-genome DEGs, showing significant changes in gene expression (magnitude of change |log2 FC| and significance -log10(p-value)) between high and low lncRNA SPACA6P-AS expressing BC samples. Upregulated genes are marked in red, downregulated in blue, and non-significant changes in gray. **B** Volcano plot of differentially expressed lncRNAs, using the same criteria to display specific lncRNA expression changes. Upregulated and downregulated lncRNAs are marked in red and blue, respectively. **C** Horizontal bar graph showing key DEGs associated with high lncRNA SPACA6P-AS expression. Bar length represents Z-score, color intensity indicates gene expression levels between high (dark) and low (light) expression groups, and significant markers included (*p < 0.05, **p < 0.01, ***p < 0.001)

In the scholarly inquiry, we undertook an in-depth analysis of data from the TCGA database using the DESeq2 package to explore genes associated with lncRNA SPACA6P-AS expression in BC. Following stringent selection criteria (adjusted p-value < 0.05, |log2 FC|> 1.5), we identified 1827 DEGs, including 1770 upregulated and 57 downregulated genes (Fig. 7). Additionally, 603 differentially expressed lncRNAs were found, with 596 upregulated and 7 downregulated.

**Fig. 8:**
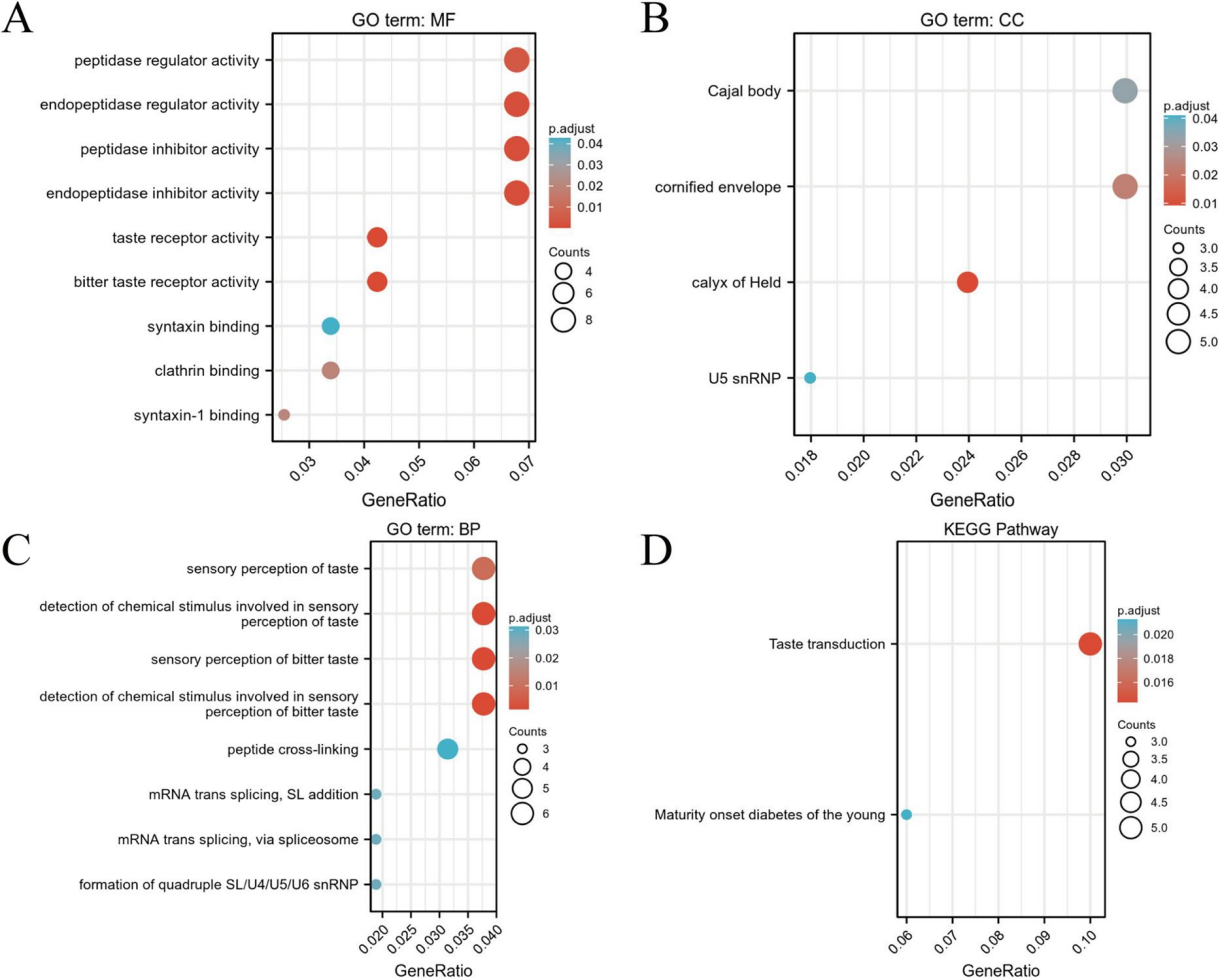
GO and KEGG pathway enrichment analysis of lncRNA SPACA6P-AS associated genes in BC. *Note*: **A** GO MF category enrichment, focusing on genes related to protease regulatory activity and taste receptor activity. **B** CC category enrichment, particularly in Cajal bodies and other cellular structure components. **C** BP category enrichment, including genes related to sensory perception of taste and mRNA transcriptional splicing. **D** KEGG pathway analysis results in notably significant enrichment in taste transduction and mature onset diabetes of the young pathways

To further understand the function and mechanisms of these DEGs in BC, we utilized the clusterProfiler tool for GO and KEGG pathway enrichment analyses. In the GO enrichment analysis, we identified three main functional categories. In the molecular function (MF) category, genes associated with protease regulatory activity, endopeptidase regulatory activity, protease inhibitor activity, endopeptidase inhibitor activity, and taste receptor activity were significantly enriched (Fig. 8A). In the cellular component (CC) aspect, genes related to Cajal bodies, cornified squamous layers, Held bodies, and U5 snRNP showed significant enrichment (Fig. 8B). In the BP category, genes linked to taste perception, identifying chemical signals in taste sensation, perception of bitterness, detection of chemical stimuli in perception of bitter taste, peptide cross-linking, mRNA transcriptional splicing, SL addition, and others were enriched (Fig. 8C).

Moreover, our KEGG enrichment analysis revealed essential pathways associated with DEGs, notably taste transduction and the mature onset diabetes of the young pathway (Fig. 8D). These results reveal the biological functions and related pathways in which SPACA6P-AS might be involved in BC and provide important clues for further research into its specific role in disease progression. Overall, through these comprehensive bioinformatics analyses, we can better understand the potential functions of lncRNA SPACA6P-AS in BC and its value as a potential therapeutic target.

### GSEA identifies signaling pathways associated with LncRNA SPACA6P-AS

The study employed GSEA to delve into the biological significance of gene sets associated with high and low expression of lncRNA SPACA6P-AS in BC. Using GSEA, we analyzed the enrichment of gene sets related to lncRNA SPACA6P-AS expression within MSigDB collections, including c2.cp.biocarta and h.all.v6.1 symbols. The analysis revealed significant signaling pathway differences with statistical significance under conditions of FDR below 0.25, with the adjusted p-value meeting the 0.05 threshold.

**Fig. 9:**
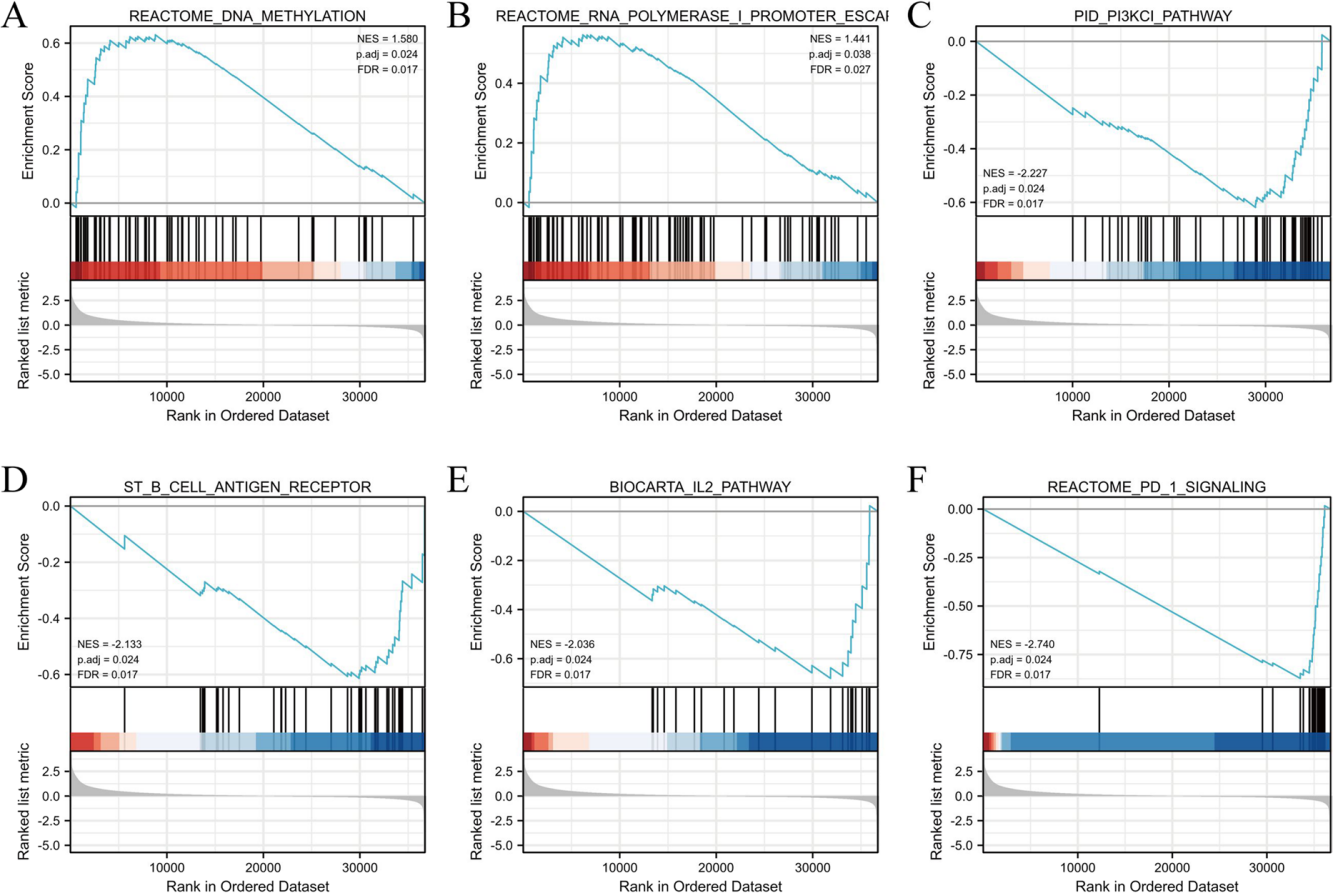
GSEA of signaling pathways associated with lncRNA SPACA6P-AS. *Note*: **A** Enrichment analysis in the REACTOME DNA methylation pathway shows the enrichment of lncRNA SPACA6P-AS high-expression associated gene sets, indicating its potential role in cancer epigenetic regulation. **B** Enrichment analysis in the REACTOME RNA polymerase I promoter escape pathway reveals lncRNA SPACA6P-AS's possible impact on gene transcription regulation. **C** Enrichment in the PID PI3KCI pathway suggests the crucial role of lncRNA SPACA6P-AS associated gene sets in vital signaling pathways for cell proliferation and survival. **D** Enrichment in the ST B cell antigen receptor pathway indicates lncRNA SPACA6P-AS's potential role in B cell maturation and function. **E** Enrichment in the BIOCARTRA IL2 pathway, related to tumor immune regulatory mechanisms. **F** Enrichment in the REACTOME PD-1 signaling pathway reveals lncRNA SPACA6P-AS's potential involvement in regulating tumor immune evasion mechanisms

We noted that lncRNA SPACA6P-AS is closely related to critical biological pathways. These pathways include DNA methylation, RNA polymerase I promoter escape, phosphoinositide 3-kinase CI (PI3KCI) pathway, B cell antigen receptor, interleukin 2 (IL2) pathway, and programmed death-1 (PD-1) signaling (Fig. 9). These findings suggest a pivotal role of lncRNA SPACA6P-AS in BC progression, particularly in cancer epigenetic regulation, signal transduction, and immune evasion. For instance, changes in DNA methylation are closely associated with tumor initiation and progression, while alterations in RNA polymerase I activity could affect transcriptional regulation. Moreover, the PI3KCI pathway plays a crucial function in cell growth and viability, commonly aberrantly activated in various cancers. Signaling through the B cell antigen receptor is vital for B cell maturation and function, and changes in the IL2 pathway and PD-1 signaling are related to tumor immune regulatory mechanisms.

### Relationship between LncRNA SPACA6P-AS expression and immune infiltration

In our study, we thoroughly analyzed immune cell infiltration characteristics in the BC tumor microenvironment, particularly examining the correlation between lncRNA SPACA6P-AS expression levels and immune cell abundance. For this purpose, we employed ssGSEA, an approach for evaluating the comparative levels of expression within distinct sets of genes in specimens. Through this method, we quantified the proportional richness of different types of immune cells in BC tumor samples and explored their correlation with lncRNA SPACA6P-AS expression using Spearman correlation analysis.

**Fig. 10:**
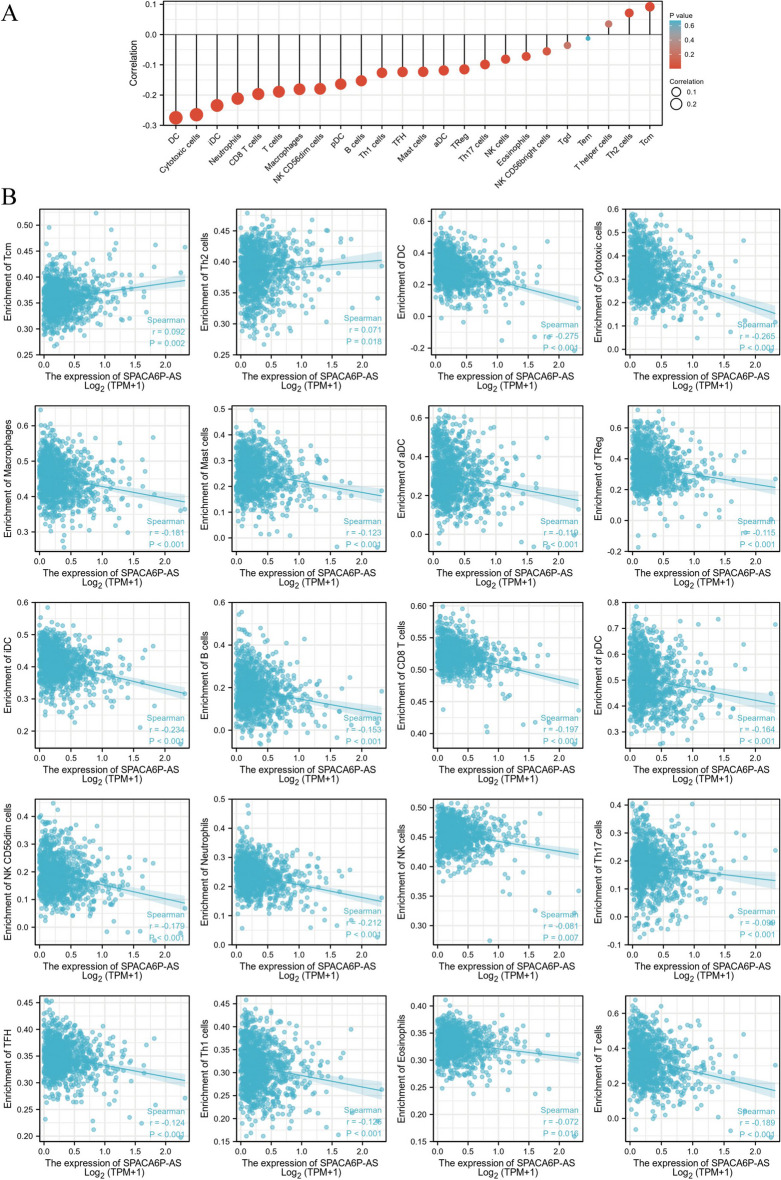
Correlation analysis of lncRNA SPACA6P-AS expression with immune cell infiltration in BC. *Note*: **A** Correlation assessment between immune cell types and lncRNA SPACA6P-AS expression levels, showing the magnitude of correlation of various immune cell types with SPACA6P-AS through DEGs analysis. **B** Detailed scatterplot matrix, each chart displaying Spearman correlation analysis results between lncRNA SPACA6P-AS expression levels and a specific type of immune cell. Deep blue points indicate the correlation between higher SPACA6P-AS expression and the relative abundance of specific immune cell types. These analyses reveal lncRNA SPACA6P-AS's key role in regulating immune cell infiltration in the BC tumor microenvironment, providing valuable immune-related biomarkers for future research

We observed significant associations between the expression of lncRNA SPACA6P-AS and the abundance of specific immune cell types (Fig. 10). For example, lncRNA SPACA6P-AS expression negatively correlated with the abundance of DCs, cytotoxic cells, iDCs, and neutrophils. This suggests that in tumor samples with higher lncRNA SPACA6P-AS expression, the abundance of these immune cells is relatively lower. Conversely, these cell types are more abundant in samples with lower lncRNA SPACA6P-AS expression.

Simultaneously, an affirmative link was observed between lncRNA SPACA6P-AS expression and specific innate immune cells, such as central memory T cells (Tcm) and T helper 2 (Th2) cells (Fig. 10). This indicates that in tumor samples with higher lncRNA SPACA6P-AS expression, the abundance of these innate immune cells is higher. These findings may suggest that lncRNA SPACA6P-AS regulates specific immune cell populations in the tumor microenvironment, potentially affecting tumor immune evasion and response.

### The promotive role of lncRNA SPACA6P-AS in BC development

**Fig. 11:**
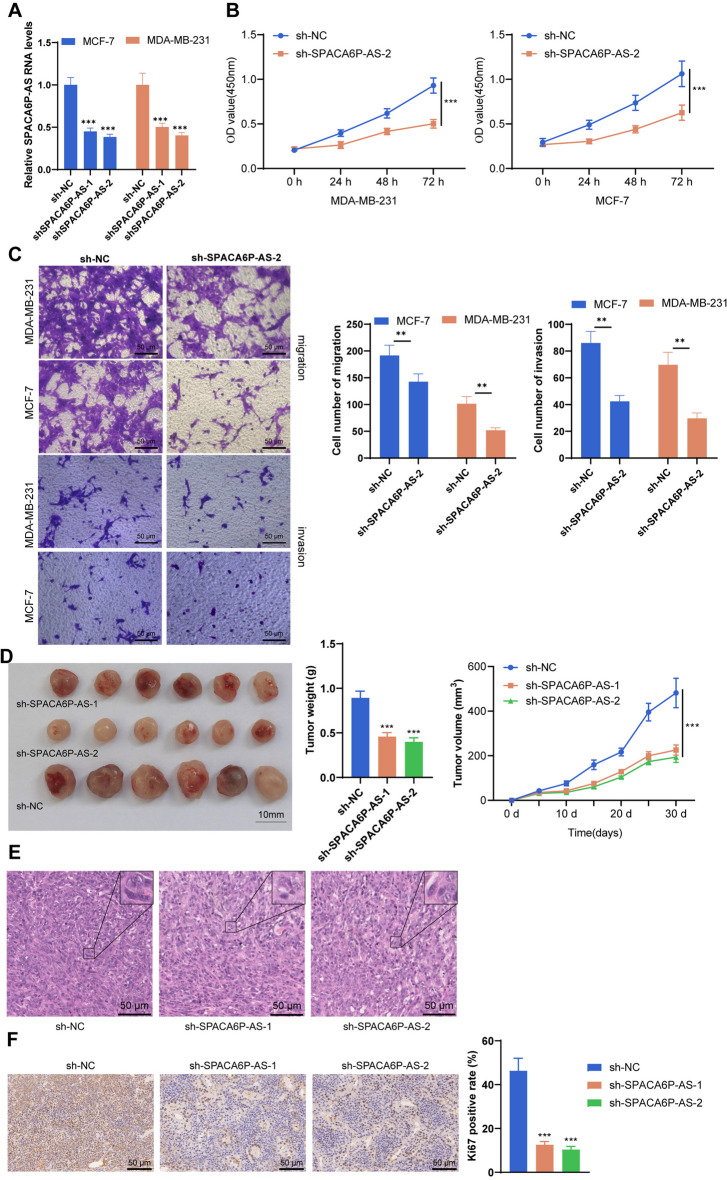
Impact of SPACA6P-AS silencing on BC cell function. *Note*: **A** Validation of SPACA6P-AS silencing in MDA-MB-231 and MCF-7 cells using siRNA technology, with expression levels measured by qPCR (*n* = 3). **B** Assessment of cell proliferation in MDA-MB-231 and MCF-7 cells post SPACA6P-AS silencing using the CCK8 assay (*n* = 3). **C** Evaluation of the impact of SPACA6P-AS silencing on the migratory and invasive abilities of MDA-MB-231 and MCF-7 cells via Transwell assays (*n* = 3). **D** Observation of tumor growth in a nude mouse model injected with MDA-MB-231 cells post SPACA6P-AS silencing (*n* = 6). **E** Assessment of the impact of SPACA6P-AS silencing on tumor tissue structure by collecting nude mouse tumor tissues and performing H&E staining (*n* = 6). **F** Ki-67 immunohistochemical staining of collected tumor tissues from nude mice to assess the impact of SPACA6P-AS silencing on cell proliferation (*n* = 6). *p < 0.05; **p < 0.01; ***p < 0.001

Through cellular and animal experiments, the significance of lncRNA SPACA6P-AS in BC initiation was rigorously studied. Initially, using specific small interfering RNA (siRNA) technology, we effectively reduced the expression of SPACA6P-AS in the BC cell lines MDA-MB-231 and MCF-7, as evidenced by quantitative real-time PCR results (Fig. 11A). Subsequent MTT assays revealed that silencing SPACA6P-AS significantly inhibited cell proliferation compared to non-target controls (Fig. 11B). Through Transwell migration and invasion assays, we further showcased that SPACA6P-AS silencing notably reduced the migratory and invasive capabilities of these BC cells (Fig. 11C). Additionally, in vivo experiments in nude mice showed that SPACA6P-AS silencing significantly inhibited tumor volume growth compared to controls (Fig. 11D). Finally, H&E staining revealed alterations in tumor tissue structure following SPACA6P-AS silencing (Fig. 11E), and a reduction in Ki-67 labeling further verified the proliferative role of SPACA6P-AS in BC cells (Fig. 11F). Collectively, the findings point towards a critical role of SPACA6P-AS in BC evolution, offering new insights for future BC therapy.

### SPACA6P-AS silencing enhances DC activation of T cells

**Fig. 12:**
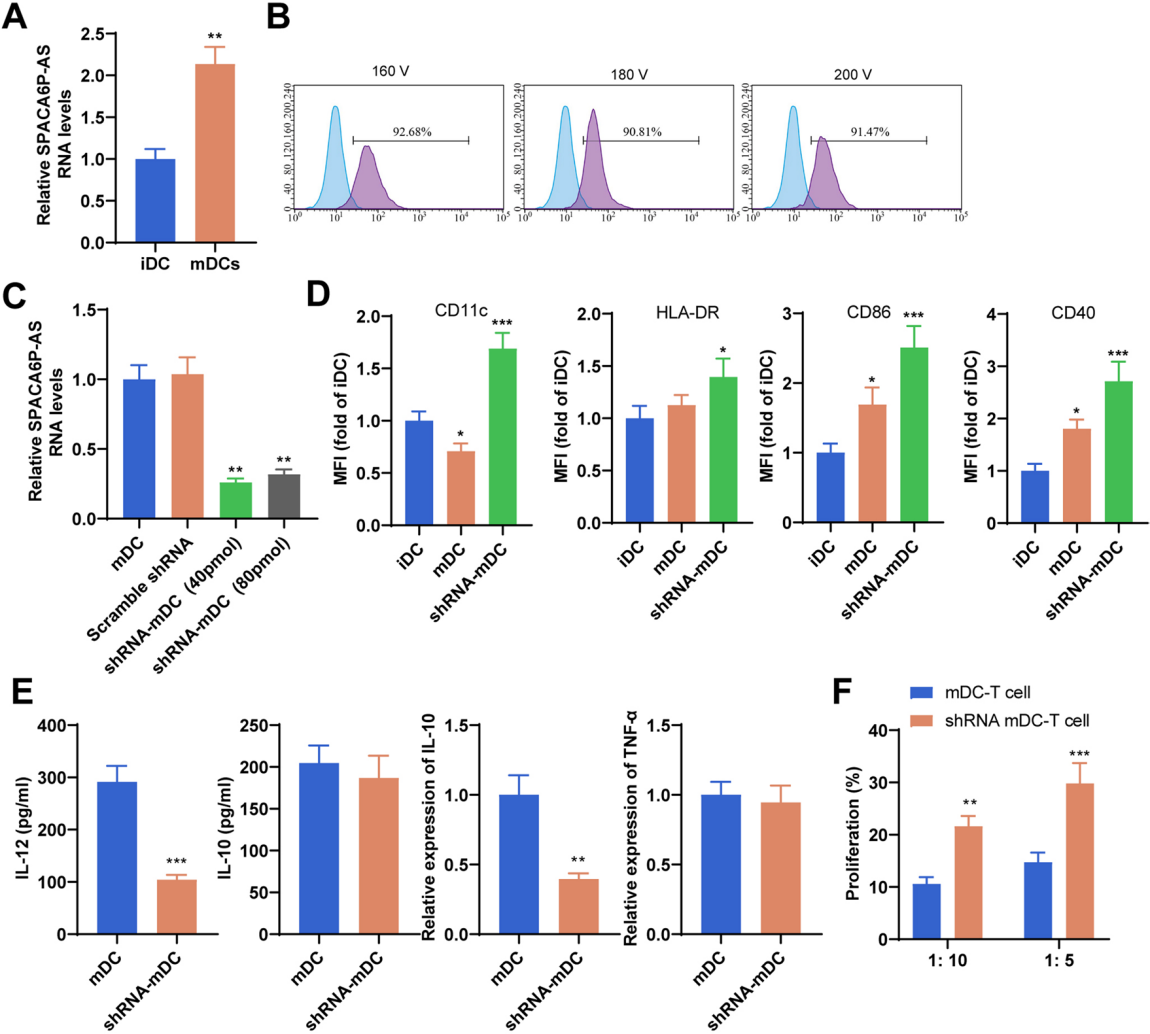
Role of SPACA6P-AS in regulating macrophage and CD3 + T cell function. *Note*: **A** Validation of siRNA-mediated SPACA6P-AS silencing in MDA-MB-231 cells using qPCR. **B** Flow cytometry analysis to assess the viability of transfected and untransfected mDCs, evaluating the impact of the transfection process on cell viability. **C** Monitoring SPACA6P-AS mRNA expression levels in mDCs post-transfection with different concentrations of SPACA6P-AS siRNA for 72 h via qPCR, determining the effective silencing concentration. **D** Flow cytometry assessment of CD11c, HLA-DR, CD40, and CD86 marker expression on iDC, mDC, and SPACA6P-AS silenced mDC surfaces, including the proportion and mean fluorescence intensity (MFI). **E** Measure IL-12 and IL-10 concentrations in cell culture supernatants and TNF-α, IL-10 mRNA expression levels using ELISA and qPCR. **F** Flow cytometry analysis to determine the proportion and proliferation level of autologous CD3 + T cells post-co-transfection of mDC and SPACA6P-AS silenced mDC. All cell experiments were replicated thrice. *p < 0.05; **p < 0.01; ***p < 0.001; ****p < 0.0001

The ssGSEA analysis unveiled an inverse relationship between SPACA6P-AS expression and immune cell infiltration, such as DCs and neutrophils, while showing a positive correlation with Tcm and Th2 cells. The study sought to methodically examine the role of siRNA-mediated silencing of lncRNA SPACA6P-AS in DC function and its impact on the proliferation and cytokine secretion of autologous CD3 + T cells. First, qPCR was used to verify SPACA6P-AS expression levels in DCs loaded with BC cell lysates, revealing significant expression changes in iDCs and mDCs post SPACA6P-AS silencing (Fig. 12A). Further analysis through flow cytometry revealed altered viability of DCs after silencing SPACA6P-AS (Fig. 12B). There were changes observed in the expression of cell surface markers CD11c and HLA-DR on DCs, indicating a potential shift in the activation state of DCs (Fig. 12E).

Subsequently, our research focused on the functional characteristics of SPACA6P-AS silenced DCs, specifically their proficiency in promoting autologous T cell proliferation and regulate cytokine secretion. Utilizing quantitative PCR and ELISA techniques, we measured key immunomodulatory factors and observed a significant reduction in IL-12 and IL-10 secretion by DCs upon SPACA6P-AS silencing (Fig. 12C and Fig. 12E).

With the aim of precisely measuring the influence of SPACA6P-AS silencing on T cell activation, we conducted co-culture experiments of DCs with autologous CD3 + T cells, and analyzed the expression of T cell surface activation markers using flow cytometry. The results revealed that SPACA6P-AS silencing notably advanced the capacity of DCs to induce T cell proliferation, confirmed through CFSE staining and flow cytometry histograms (Fig. 12D and Fig. 12F). Additionally, we observed an upregulation in the expression of DC surface co-stimulatory molecules CD86 and CD40 upon SPACA6P-AS silencing, which are vital for T cell activation (Fig. 12D).

## Discussion

Within the scope of this investigation, through analysis of RNA sequencing and clinical data from 1083 BC patients in the TCGA database, we confirmed a significant upregulation of SPACA6P-AS in BC tissues, closely correlated with unfavorable outcome. This finding is consistent with previous studies (Woolston 2015; Michaels et al. 2023; Ibrahim et al. 2018; Zavala et al. 2019; Schnitt 2010), further underscoring the role of lncRNA in BC development (Li et al. 2022b; Shi et al. 2023; Hu et al. 2023). For the first time, this study validated the differential expression of SPACA6P-AS in BC and normal tissues in the TCGA and GTEx databases, revealing its potential as a biomarker to distinguish normal from neoplastic tissues.

Through analysis of the BP and signaling pathways concerning SPACA6P-AS, this study further deepened our understanding of its function. Our enrichment analysis revealed associations of SPACA6P-AS with various BP and signaling pathways, potentially impacting immune regulation, growth and proliferation, glucose metabolism, and DNA methylation among others. These processes play crucial roles in tumor development and metastasis, especially concerning the control of cell migration and invasion capacities. Furthermore, this study also uncovered potential effects of SPACA6P-AS on important signaling pathways such as PI3K/Akt, B cell receptor, IL2 pathway, and PD-1 signaling.

In this study, we found an inverse relationship between the expression of SPACA6P-AS and the infiltration of DCs and neutrophils, and a positive correlation with Tcm and Th2 cells. This discovery reveals the potential role of SPACA6P-AS in regulating the tumor immune microenvironment. Through ssGSEA and Spearman correlation analysis, we further investigated the correlation between SPACA6P-AS expression and the degree of infiltration by immune cells, emphasizing its importance in immune modulation. This contrasts with previous studies on the function of other long non-coding RNAs in immune regulation, highlighting the possible role of SPACA6P-AS in promoting the activation of specific immune cells.

Findings from experiments conducted in vitro and in vivo indicate that silencing SPACA6P-AS through shRNA significantly reduces the proliferation, migration, and invasion abilities of BC cells. Moreover, DCs with silenced SPACA6P-AS show enhanced functionality in promoting the proliferation of autologous CD3 + T cells and cytokine secretion, revealing a potential immunosuppressive role of SPACA6P-AS in DC function. The immunomodulatory impact of SPACA6P-AS on DCs and T cell activation is uncovered in this investigation for the first time. It suggests that inhibiting SPACA6P-AS in DCs could be an effective strategy to enhance DC activation of T cell responses and improve the efficacy of cell-based therapies reliant on DCs. These findings not only put forward a unique viewpoint on the significance of DCs in the BC microenvironment but also provide crucial molecular targets for the development of novel DC-based immunotherapy strategies.

Our research reveals that SPACA6P-AS stands out as a unique therapeutic target for BC. Compared to other potential therapeutic targets mentioned in existing literature, SPACA6P-AS displays unique potential in regulating the immune microenvironment. Additionally, our research indicates that targeting SPACA6P-AS can impact the proliferation, migration, and invasion capabilities of BC cells. This discovery not only offers new treatment strategies but also opens avenues for personalized BC therapy.

The discovery of lncRNA SPACA6P-AS in BC holds significant clinical value. Firstly, the expression levels of SPACA6P-AS can be utilized as a biomarker for BC, aiding in the early-stage differentiation between normal and neoplastic tissues, thereby enhancing diagnostic accuracy. Furthermore, due to the close association between SPACA6P-AS expression in BC and patient prognosis, it can serve as a prognostic indicator, aiding clinicians in better assessing disease progression and treatment responses. Regarding treatment, as a potential target, SPACA6P-AS offers possibilities for the creation of innovative targeted treatments for BC. Particularly in immune therapy, its close correlation with immune cell infiltration and activation suggests it may be crucial in enhancing the effectiveness of BC immune therapies. Lastly, the construction of a prognostic nomogram based on SPACA6P-AS aids in providing personalized treatment plans and prognostic predictions for each BC patient, thereby improving treatment outcomes and quality of life.

Our study combines bioinformatics analysis and experimental validation, a methodology not commonly seen in current BC research. Through the extensive analysis of data from the TCGA database and validation through in vitro cell and animal experiments, we have strengthened the evidence supporting our research findings. Compared to studies relying solely on single experimental methods, this combination not only enhances the reliability of the research but also increases the generalizability of the discoveries. However, our study has certain limitations. Firstly, as the data primarily stem from public databases, there may be sample selection biases and issues of data incompleteness. For example, samples in the TCGA database may not fully represent the diversity of all BC patients. Secondly, our study did not delve deeply into the specific molecular mechanisms of SPACA6P-AS, limiting a comprehensive understanding of its role in BC development. Additionally, despite some validation with clinical samples in our study, there is still a lack of large-scale preclinical and clinical trials to confirm the effectiveness and safety of SPACA6P-AS as a therapeutic target. Lastly, our study design is retrospective, which may introduce retrospective analysis biases, highlighting the need for future prospective studies to validate these findings.

To address these limitations, future research should focus on several areas. Firstly, broader and more diverse population studies are needed, encompassing different racial groups, BC subtypes, and patients with varying treatment responses, to enhance the universality and translatability of research results. Secondly, in-depth experimental studies essential to delve into the precise molecular pathways associated with SPACA6P-AS in BC, particularly its influence on the tumor microenvironment and immune cell infiltration and activation. Additionally, conducting more preclinical and clinical trials, especially regarding the potential of SPACA6P-AS as a targeted therapy and immunotherapy target, will be crucial. These studies will not only validate the clinical application value of SPACA6P-AS but also drive innovation and development in BC treatment strategies. Ultimately, the design of prospective studies will aid in better understanding the role of SPACA6P-AS in BC and provide more precise guidance for future treatments.

## Conclusion

**Fig. 13:**
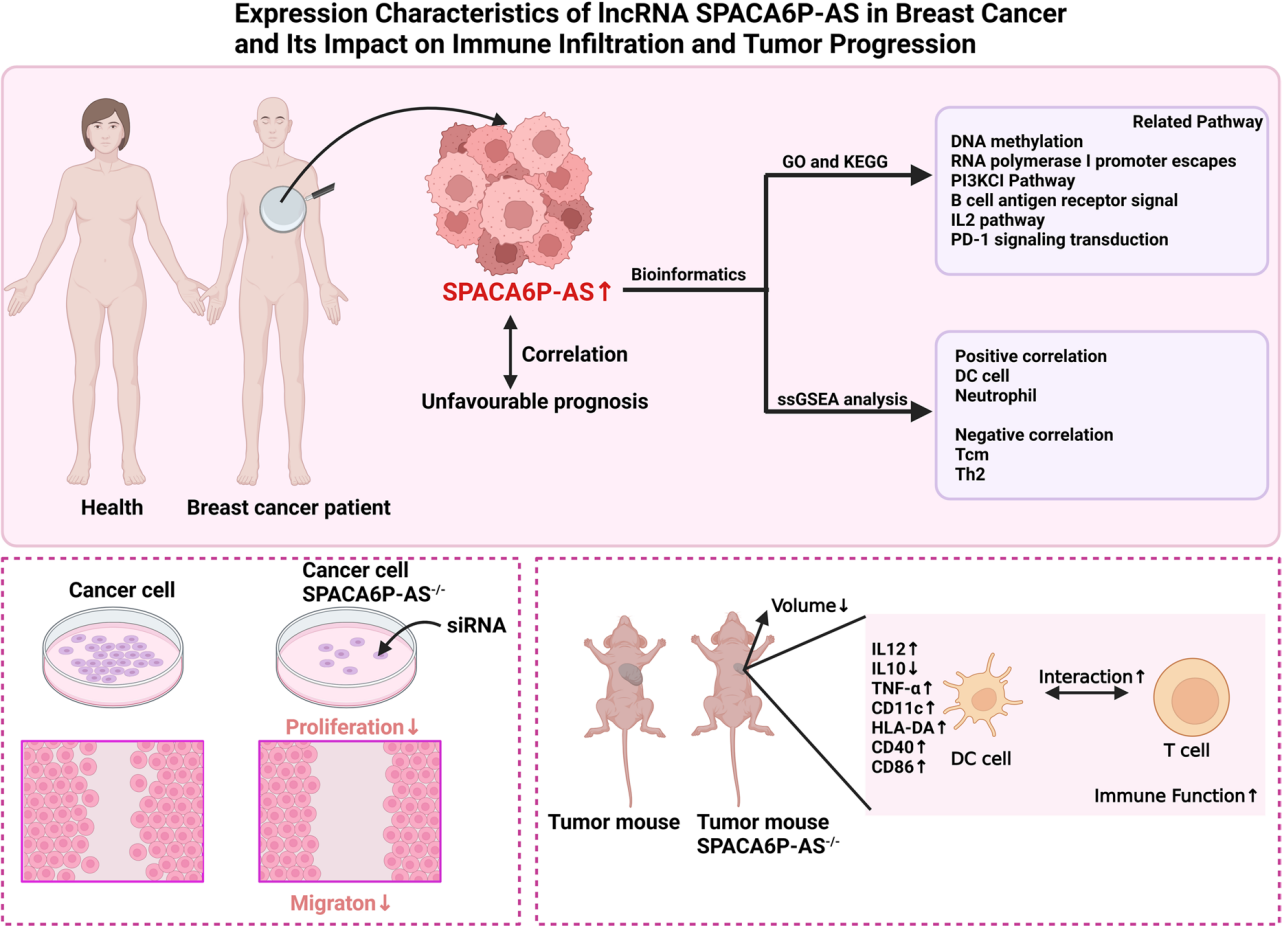
Expression characteristics of lncRNA SPACA6P-AS in BC and its impact on immune infiltration and tumor progression

This study comprehensively analyzed the role and impact of the lncRNA SPACA6P-AS in BC development, providing in-depth bioinformatics and clinical data support (Fig. 13). These discoveries underscore the possible involvement of SPACA6P-AS in BC development and offer crucial scientific groundwork for future clinical applications and treatment strategies. In conclusion, this research furnishes robust evidence for utilizing SPACA6P-AS as a point of focus for BC therapy initiatives and lays the foundation for further functional studies and clinical trials.

## Author' contributions

WJF, YLJ and LJZ planned the experiments; YHOY, HLL, YBT AND LQL performed the experiments, prepared the figures and analysed the data; LJOY performed some of the experiments; LMX, YRT, YHL contributed to drafting the manuscript All authors have read and approved the final submitted manuscript.

## Supplementary Information

Below is the link to the electronic supplementary material.

Supplementary file1 (DOCX 15 KB)

## Data Availability

The datasets generated and analyzed during the current study are available from the corresponding author upon reasonable request. Without restrictions, the raw data could also be obtained from online databases, including the TCGA, and GEO.
