# Supplementary material for: SPACA6P-AS: a trailblazer in breast cancer pathobiology and therapeutics
Source: Cell Biol Toxicol. 2024 Jun 26;40(1):49. doi: 10.1007/s10565-024-09870-9 (PMC11208203; doi:10.1007/s10565-024-09870-9)
Supplement: Supplementary file 1 — Supplementary file1 (DOCX 15 KB) [file 10565_2024_9870_MOESM1_ESM.docx]

**Table S1. Primer sequences for real-time quantitative PCR amplification of lncRNA SPACA6P-AS and GAPDH.**

| **Gene** | **LncRNA SPACA6P-AS** |
| --- | --- |
| LncRNA SPACA6P-AS | Forward: 5'-AGGTCAGGTTTCAGTTGGTGG-3' |
|  | Reverse: 5'-GCACACCATGTTGCCAGTCT-3' |
| GAPDH | Forward: 5'-GACAGTCAGCCGCATCTTCT-3' |
|  | Reverse: 5'-GCGCCCAATACGACCAAATC-3' |
